# A Hybrid Deep Learning Framework for Automated Dental Disorder Diagnosis from X-Ray Images

**DOI:** 10.3390/jcm15031076

**Published:** 2026-01-29

**Authors:** A. A. Abd El-Aziz, Mohammed Elmogy, Mahmood A. Mahmood, Sameh Abd El-Ghany

**Affiliations:** 1 Department of Information Systems, College of Computer and Information Sciences, Jouf University, Sakaka 72388, Saudi Arabia; mamahmood@ju.edu.sa (M.A.M.); saabdelwahab@ju.edu.sa (S.A.E.-G.); 2 Information Technology Department, Faculty of Computers and Information, Mansoura University, Mansoura 35516, Egypt; melmogy@mans.edu.eg

**Keywords:** dental disease, deep learning, radiography X-rays, long short-term memory, histogram of oriented gradients, shifted window transformer

## Abstract

**Background:** Dental disorders, such as cavities, periodontal disease, and periapical infections, remain major global health issues, often resulting in pain, tooth loss, and systemic complications if not identified early. Traditional diagnostic methods rely heavily on visual inspection and manual interpretation of panoramic X-ray images by dental professionals, making them time-consuming, subjective, and less accessible in resource-limited settings. **Objectives**: Accurate and timely diagnosis is vital for effective treatment and prevention of disease progression, reducing healthcare costs and patient discomfort. Recent advances in deep learning (DL) have demonstrated remarkable potential to automate and improve the precision of dental diagnostics by objectively analyzing panoramic, periapical, and bitewing X-rays. **Methods:** In this research, a hybrid feature-fusion framework is proposed. It integrates handcrafted Histogram of Oriented Gradients (HOG) features with deep representations from DenseNet-201 and the Shifted Window (Swin) Transformer models. Sequential dependencies among the fused features were learned utilizing the Long Short-Term Memory (LSTM) classifier. The framework was evaluated on the Dental Radiography Analysis and Diagnosis (DRAD) dataset following preprocessing steps, including resizing, normalization, Contrast Limited Adaptive Histogram Equalization (CLAHE) enhancement, and image cropping. **Results**: The proposed LSTM-based hybrid model achieved 96.47% accuracy, 91.76% specificity, 94.92% precision, 91.76% recall, and 93.14% F1-score. **Conclusions**: The proposed framework offers flexibility, interpretability, and strong empirical performance, making it suitable for various image-based recognition applications and serving as a reproducible framework for future research on hybrid feature fusion and sequence-based classification.

## 1. Introduction

Dental diseases rank among the most prevalent health issues worldwide, significantly impacting a person’s overall health and quality of life. The mouth functions not only as the gateway to the digestive tract but also plays a crucial role in communication, social interactions, and aesthetics [1]. Failing to maintain proper oral hygiene leads to the buildup of bacterial plaque on teeth, which gradually wears down enamel and can lead to cavities. If these issues remain unaddressed, they can advance to periodontal disease, complications involving the jawbone, and potentially systemic health concerns. These effects underscore the critical link between oral health and overall wellness, underscoring the importance of dental care in maintaining good health [2].

In addition to causing pain, tooth loss, and diminished quality of life, dental diseases can have extensive implications for systemic health. Studies indicate that issues like caries and gum disease are linked to severe conditions, such as heart disease and heart attacks. The repercussions of inadequate oral health affect individuals across various age groups and socioeconomic statuses, often leading to significant pain, distress, and, in severe cases, life-threatening infections. To reduce these risks, it is essential to identify dental diseases early through radiographic imaging and other diagnostic methods, as prompt intervention can prevent complications and enhance long-term health outcomes [3].

Improvements in medical technology and digital systems have profoundly transformed healthcare organizations, and this shift is also influencing dentistry. Recent studies show that oral health can serve as an important diagnostic indicator of diabetes, cardiovascular disease, and other systemic conditions. These findings underscore that oral health is not a separate issue but a vital aspect of holistic healthcare, necessitating the adoption of contemporary diagnostic tools and techniques [3].

Imaging plays a crucial role in dentistry, allowing professionals to detect issues that may not be apparent during a clinical exam. Techniques, such as X-rays, computed tomography (CT) scans, and magnetic resonance imaging (MRI), provide comprehensive insights into the condition of teeth, bones, and soft tissues [4]. These tools enhance the precision of diagnoses and treatments, ensuring better patient outcomes. Among current imaging exams, radiographs are the most common in dentistry, requested to identify various pathologies such as cavities, periodontal disease, impacted teeth, and bone infections, and to track the progress of dental treatments [4,5]. Radiological assessments in dentistry aid professionals by revealing the configuration of dental bones to evaluate impacted teeth, bone irregularities, cysts, tumors, infections, and fractures [6].

Dentists often use radiographic X-ray images to assess the entire dental structure prior to upcoming treatments. A radiological X-ray scan is a diagnostic tool in dentistry that evaluates the health of a patient’s teeth, gums, jaws, and bone structure, aiding in the identification of oral diseases. There are two primary categories of radiological X-rays used in dental practice: **intraoral**, where the film is positioned inside the mouth, and **extraoral**, where the patient’s face is situated between the radiographic film and the X-ray source. The three main types of dental radiological X-rays include **extraoral** panoramic radiography (commonly referred to as a panoramic X-ray or orthopantomography), **intraoral** bitewing radiography (also known as bitewing X-rays), and **periapical** intraoral radiography [7].

While dentists play a crucial role in identifying dental problems, the manual analysis of X-ray images can be complex. For instance, subjective interpretations can lead to inconsistencies in identifying cavities among different observers. Additionally, factors such as radiograph quality, viewing conditions, the dentist’s preconceived notions, and the time spent on each examination can affect detection accuracy [8]. Additionally, mistakes made during manual analysis can lead to flawed predictions. Furthermore, manual clinical assessments are often lengthy, labor-intensive, and monotonous [9].

Recent advancements in digital technologies within healthcare have significantly enhanced the field of dentistry. Artificial intelligence (AI) has played a critical role in this transformation. AI-driven systems in oral and dental health are used not only to analyze radiological images but also to provide diagnostic recommendations, categorize structures with anomalies, predict treatment outcomes, and facilitate early disease identification [10]. AI can analyze data more quickly and consistently than traditional methods, thereby improving the accuracy of dental disease diagnosis. This technology also streamlines clinical processes and enhances patient satisfaction. Additionally, AI enhances the reliability of diagnostic procedures by identifying subtle details that the human eye may miss [11].

The application of AI in dental radiology offers considerable advantages in both clinical and educational settings. AI-driven systems assist dental students in navigating the challenges of diagnostic procedures, thereby enhancing their proficiency in handling intricate cases. These technologies can enable a more precise, focused diagnostic approach by minimizing distractions from external factors. Furthermore, AI analysis tools enable both dentists and students to improve their time management and enhance their clinical learning experiences [12].

DL algorithms, especially convolutional neural networks (CNNs), can autonomously analyze dental radiographs (including X-rays, CBCT scans, and panoramic images) to identify irregularities such as cavities, periodontal disease, impacted teeth, and jaw lesions. These DL models can achieve accuracy levels comparable to those of seasoned dentists, minimizing human error and variability across examiners [12]. DL can recognize intricate patterns in dental images that may not be apparent to the human eye, facilitating early identification of conditions such as dental decay, periapical lesions, and bone deterioration. Timely diagnosis improves treatment outcomes and reduces healthcare costs. Dental clinics generate large volumes of imaging data. DL models can process thousands of images rapidly, providing fast diagnostic support, prioritizing urgent cases, and assisting dentists in decision-making [4].

In this paper, we present a novel hybrid DL framework that integrates HOG, a handcrafted descriptor, with DenseNet-201 and the Swin Transformer for feature extraction, thereby improving classification performance. HOG, DenseNet-201, and Swin Transformer collaborate to effectively capture complementary information, encompassing fine-grained low-level spatial characteristics as well as rich high-level semantic representations. These features are extracted simultaneously from HOG, DenseNet-201, and Swin. The extracted features are fused to create a unified representation. Feature fusion is executed sequentially, beginning with the combination of handcrafted features from HOG and deep convolutional features from DenseNet-201. This fused representation is then integrated with high-level contextual features extracted by the Swin Transformer. This hierarchical approach enriches low-level texture information with deep semantic and global contextual insights. Finally, the LSTM network is used for classification, leveraging its ability to model sequential dependencies within the fused features. The fused features are processed by a trainable fusion layer with fully connected (FC) layers and nonlinear activation functions. This process allows the LSTM network to learn optimal fusion weights end-to-end, adapting the weighting of each feature source based on its relevance.

An ablation study is included to assess the contribution of each feature set—transformers only, CNNs only, dual-fusion, and full triple-fusion—under consistent experimental conditions. For classification, the system uses an LSTM classifier instead of traditional machine learning (ML) methods, enabling temporal modeling of feature embeddings and improving separability in high-dimensional spaces. Extensive experiments using 5-fold cross-validation show the robustness of the pipeline and underscore the synergistic effect of combining deep and handcrafted features. Results include average precision, recall, specificity, accuracy, and F1-scores, providing a thorough evaluation of model performance and feature impact.

We performed experiments on the DRAD dataset [13] for multiclass classification, with preprocessing steps including resizing, normalization, CLAHE enhancement, and image cropping. A comprehensive statistical evaluation and comparison with three top DL models—DenseNet-121, VGG-16, and MobileNet—demonstrated that the proposed hybrid DL framework achieved superior performance. Below are the key contributions of our research:

- We introduced a new hybrid DL framework that combines handcrafted and deep feature extraction techniques, integrating HOG, DenseNet-201, and the Swin Transformer. The hybrid DL framework demonstrated that combining diverse handcrafted and deep representations significantly outperformed typical single-model, uniform-feature-based methods used in dental X-ray analysis.
- The proposed framework demonstrated that using dependency-aware sequence modeling of fused feature embeddings leads to better multiclass diagnostic performance than conventional static classifiers and traditional machine learning models.
- We conducted a comparative analysis against DenseNet-121, VGG-16, and MobileNet and found that our proposed hybrid framework achieved superior results across all major evaluation metrics.
- Our hybrid framework showed remarkable accuracy in detecting dental diseases while using minimal time and resources. Additionally, it proved valuable in pathology, enabling timely, personalized patient treatment.
- We conducted an ablation study to confirm the model’s architectural and design decisions adopted during its development.
- Compared to four ML models, the LSTM model achieved superior performance in a multi-class classification task, attaining 96.47% accuracy, 91.76% specificity, 94.92% precision, 91.76% recall, and 93.14% F1-score.

The remainder of this research is structured as follows. In Section 2, the current literature on diagnostic systems for dental disease is examined. In Section 3, we describe the DRAD dataset and explain the workflow of the proposed framework. In Section 4, we present our experimental outcomes of the proposed hybrid DL framework. Lastly, in Section 5, we wrap up and show a summary of the results.

## 2. Literature Review

Diagnosis of dental diseases is a significant focus of research in medical image analysis. Many studies tackle this topic from diverse perspectives. For example, Alsakar et al. [2] introduced an ML and DL-based system designed to assist in the early diagnosis of dental diseases. The proposed diagnostic framework utilized X-ray imaging and features a comprehensive pre-processing phase that employs image normalization and adaptive histogram equalization to enhance image quality and minimize variability. A dual-stream methodology was implemented for feature extraction, leveraging the strengths of the Swin Transformer to capture long-range dependencies and global context, and MobileNet-V2 for efficient local feature extraction. The fused features created a detailed representation of dental anomalies. To achieve reliable, broadly applicable classification results, a bagging ensemble classifier was used at the final stage. The proposed model was evaluated on a benchmark dataset of dental radiographs and achieved 95.7% precision, 95.4% sensitivity, 95.7% specificity, 95.5% F1-score, and 95.6% accuracy.

Yüksel et al. [5] introduced a novel and robust DL framework, DENTECT, designed to quickly identify five distinct dental treatment methods while simultaneously numbering teeth according to the FDI notation in panoramic X-ray images. DENTECT is the first system to concentrate on recognizing multiple dental procedures, specifically periapical lesion therapy, fillings, root canal treatment (RCT), surgical extraction, and conventional extraction, all of which were accurately positioned within their respective borders and tooth numbers. Even though DENTECT was trained using only 1005 images, the expert-provided annotations yield commendable results for both treatment identification and enumeration. The framework achieved an average precision (AP) of 89.4% for enumeration and 59.0% for treatment identification.

You et al. [6] utilized a CNN, trained on 886 intraoral images of primary teeth. To assess clinical applicability, an additional 98 intraoral images were evaluated by the AI model. These tooth images were captured using a digital camera. An experienced pediatric dentist reviewed the images and highlighted the areas with plaque. A plaque-disclosing agent was subsequently used to identify these regions. After one week, the dentist re-examined the 98 digital photos to reassess the identified plaque areas and ensure consistency with the manual diagnosis. Furthermore, 102 intraoral images of primary teeth were annotated to indicate the plaque areas identified by both the AI model and the dentist, allowing for a comparison of the diagnostic accuracy of each method using lower-resolution images. The mean intersection-over-union (mIoU) metric was used to evaluate detection precision. The mIoU for the AI model in detecting plaque on the analyzed tooth images was 0.726 ± 0.165. For the dentist, the MIoU was 0.695 ± 0.269 during the initial assessment of the 98 digital photos and 0.689 ± 0.253 after one week. The AI model achieved a superior mIoU of 0.736 ± 0.174, with no change after 1 week. When both the dentist and the AI model evaluated the 102 intraoral images, the mIoU was 0.652 ± 0.195 for the dentist and 0.724 ± 0.159 for the model.

Baydar et al. [14] analyzed 500 bite-wing radiographs from the radiographic archive at Eskişehir Osmangazi University, Faculty of Dentistry, Department of Oral and Maxillofacial Radiology. The CranioCatch labeling program (CranioCatch, Eskisehir, Turkey) was utilized to label images with information on tooth decay, crowns, pulp, restoration materials, and root-filling materials, employing a segmentation technique for five distinct diagnoses. They proposed an AI model using the U-Net architecture. The study yielded the following performance metrics: for caries, the F1 score, sensitivity, and precision were 88.18%, 82.35%, and 94.91%, respectively; for crowns, the scores were 96.29%, 92.85%, and 100%; for pulp, they were 96.31%, 98.43%, and 94.29%; for restoration material, the scores were 97.14%, 96.22%, and 98.07%; and for root filling material, they were 97.22%, 94.59%, and 100%.

Lee et al. [15] assessed the effectiveness of deep CNNs in identifying and diagnosing dental caries using periapical radiographs. A total of 3000 periapical radiographic images were split into a training and validation set (2400 images [80%]) and a test set (600 images [20%]). For preprocessing and transfer learning, a pre-trained GoogLeNet (Inception v3) CNN model was used. Various metrics were computed to evaluate the detection and diagnostic capabilities of the deep CNN algorithm, including diagnostic accuracy, sensitivity, specificity, positive predictive value (PPV), negative predictive value (NPV), the receiver operating characteristic (ROC) curve, and the area under the curve (AUC). The diagnostic accuracies for the premolar, molar, and combined premolar and molar models were 89.0% (80.4–93.3), 88.0% (79.2–93.1), and 82.0% (75.5–87.1), respectively. The deep CNN algorithm yielded an AUC of 0.917 (95% CI 0.860–0.975) for the premolar model, 0.890 (95% CI 0.819–0.961) for the molar model, and 0.845 (95% CI 0.790–0.901) for the combined model. The premolar model achieved the highest AUC, which was significantly greater than that of the other models (*p* < 0.001).

Deng et al. [16] introduced a CNN architecture that leverages DL for the classification of four types of tooth X-ray images: normal teeth, implants, fillings, and abnormal teeth (cavities). The neural network architecture was crucial to this research, prompting multiple modifications to both the CNN design and the test set parameters to optimize performance. Initial findings revealed that the detection accuracy rates for the four categories were as follows: normal teeth at 87%, implants and fillings at 98%, and cavities at 89%. Overall, the authors achieved an average accuracy of 93.04%.

Abdalla-Aslan et al. [17] created a computer vision algorithm utilizing AI to automatically identify and categorize various dental restorations in panoramic radiographs. A total of 738 dental restorations across 83 anonymized panoramic images were examined. The images were automatically cropped to focus on the areas of interest, specifically the maxillary and mandibular alveolar ridges. Subsequently, the restorations were segmented using a local adaptive thresholding technique. These segmented restorations were then classified into 11 distinct categories, and the algorithm was trained to recognize them. Numerical characteristics derived from the shape and distribution of gray-level values were extracted to facilitate classification of the restorations into different categories. Ultimately, a Cubic Support Vector Machine algorithm, paired with Error-Correcting Output Codes, was employed for multiclass classification based on these features, using cross-validation. The algorithm successfully detected 94.6% of the restorations. The classification process eliminated all incorrect markings, resulting in 90.5% of the restorations being accurately identified in the images. The overall classification accuracy in distinguishing the true restoration categories was 93.6%.

Ghaznavi Bidgoli et al. [18] compiled a standardized dataset of panoramic radiographs depicting jaws and teeth. A deep neural network was used to classify teeth into four categories: healthy, decayed, root-canaled, and restored. The dataset was segmented into five groups to assess the performance of the proposed method. Various evaluation metrics, confusion matrices, and precision rates were calculated for each group. The final average precision across the five groups was 92%. Additionally, the trained network’s outputs were compared with those of AlexNet and VGGNet16.

Jaiswal et al. [19] used a custom dataset comprising 500 images illustrating six dental diseases across 46 potential combinations. The diseases include tooth wear, periapical lesions, periodontitis, tooth decay, missing teeth, and impacted teeth. The proposed system employed transfer learning, integrating various pre-trained networks, including ResNet50-V2, ResNet101-V2, MobileNet-V3Large, MobileNet-V3Small, MobileNet, EfficientNet-B0, EfficientNet-B1, and EfficientNet-B2, along with XGBoost to produce final predictions. The authors split the dataset into a training set (80%) and a test set (20%). Results indicated that the model achieved detection accuracies of 91.8%, 92.2%, 92.4%, 93.2%, 91.6%, and 90.8% for tooth wear, periapical lesions, periodontitis, tooth decay, missing teeth, and impacted teeth, respectively.

Krois et al. [20] proposed deep CNNs to identify periodontal bone loss (PBL) in panoramic dental radiographs. A dataset of 2001 image segments was created from these radiographs, with the reference test being the percentage of PBL measured. A deep feed-forward CNN was trained and evaluated using 10-fold group shuffling. The authors optimized the model’s architecture and hyperparameters using a grid search. The final architecture consisted of a seven-layer deep neural network, containing a total of 4,299,651 weights. For comparison, six dentists evaluated the same image segments for PBL. Across 10 validation folds, the CNN achieved an average standard deviation (SD) classification accuracy of 0.81 (0.02). The mean (SD) sensitivity and specificity were recorded at 0.81 (0.04) and 0.81 (0.05), respectively. In contrast, the dentists’ accuracy averaged (SD) 0.76 (0.06), indicating that the CNN did not show a statistically significant advantage over the dentists (*p* = 0.067/*t*-test). The dentists’ mean sensitivity and specificity were 0.92 (0.02) and 0.63 (0.14), respectively. A CNN trained on a limited number of radiographic image segments demonstrated discrimination capabilities comparable to those of dentists when assessing PBL in panoramic radiographs.

The limitations of the state-of-the-art are as follows:

1. Dependence on Single Sources or Similar Features: Many studies rely primarily on CNN-based deep features (such as GoogLeNet, U-Net, ResNet, VGG, and MobileNet) or handcrafted descriptors, failing to effectively integrate complementary handcrafted and deep representations. This prevents the model from effectively capturing both fine-grained textures and overarching semantic information simultaneously. However, we integrated diverse feature sources by combining HOG for low-level texture and edge information, DenseNet-201 for hierarchical local and mid-level semantic representations, and Swin Transformer for global contextual and long-range dependency modeling. This triple-feature fusion strategy ensures the capture of complementary information, addressing the limitations of single-source approaches.
2. Limited Use of Global Context and Long-Range Relationships: Although transformer-based architectures have been explored in a few works (e.g., Swin Transformer), most current approaches rely on CNNs that focus primarily on local receptive fields, which may not be sufficient for modeling global structural relationships in complex dental radiographs. The inclusion of the Swin Transformer explicitly addressed this limitation by modeling long-range spatial dependencies and global context via shifted-window attention, while maintaining computational efficiency. Its fusion with CNN and handcrafted features enabled balanced local–global representation learning.
3. Lack of Sequential or Dependency-Aware Classification: Most studies use conventional classifiers (like softmax layers, SVMs, ensemble learners) that treat features independently. Few studies consider inter-feature dependencies or sequential relationships, which are essential when combining heterogeneous feature embeddings. In our research, an LSTM-based classifier was employed to model sequential dependencies among fused feature embeddings, enabling the framework to learn correlations and ordering relationships within high-dimensional feature spaces. This design enhances class separability and addresses the limitation of treating features independently.
4. Task-Specific and Limited Clinical Scope: Several frameworks are tailored to specific tasks, such as plaque detection, periodontal bone loss assessment, or treatment enumeration. While effective, these approaches cannot generalize across various dental diseases and imaging conditions. In our research, we designed the proposed framework as a general-purpose, multiclass diagnostic pipeline, evaluated on the DRAD dataset and applicable to diverse dental disease categories. Its modular design allows straightforward adaptation to.
5. In previous studies, the researchers did not conduct an ablation study. Conversely, we conducted this investigation to understand how each element or feature of our proposed hybrid framework affects performance, systematically altering or removing components and evaluating their effects.
6. In previous studies, researchers primarily focused on performance metrics such as specificity, accuracy, recall, precision, and F1-score, without conducting any statistical analysis. In contrast, our study includes a statistical test to rigorously confirm the superiority of the proposed hybrid DL framework compared to existing state-of-the-art methods.

## 3. Framework Architecture and Methods

### 3.1. Dental Radiography Analysis and Diagnosis Dataset

**Figure 1:**
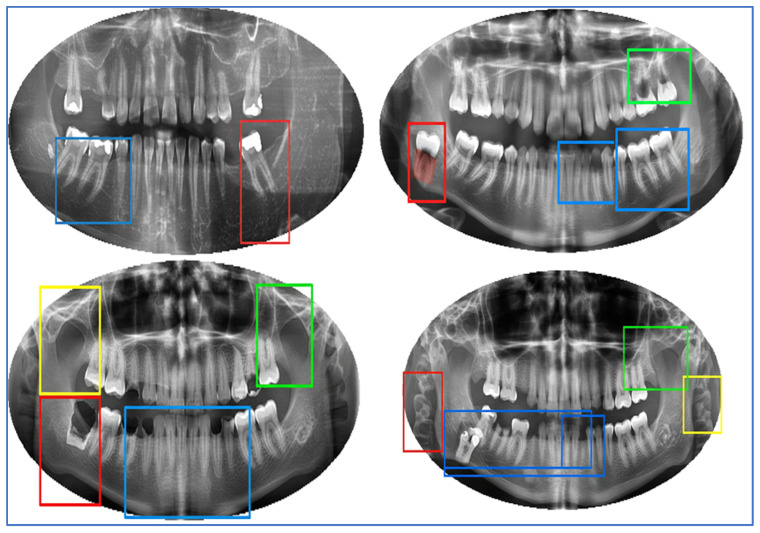
DRAD dataset’s samples [2].

The DRAD dataset focused on dental radiography analysis and diagnosis. The dataset consists of 1272 X-ray images, likely including intraoral or panoramic views, for detecting and classifying conditions such as implants, cavities, fillings, and impacted teeth. Notably, specific images may exhibit multiple classifications simultaneously. The dataset was divided into three parts: 37.5% for the training set (478 images), 30.8% for the testing set (392 images), and 31.8% for the validation set (402 images) [13]. Figure 1 shows samples of the DRAD dataset, highlighting expert-annotated regions of interest. Red boxes indicate pathological regions of the tooth, such as dental caries or abnormal structures. Blue boxes denote normal or healthy teeth. Green boxes highlight impacted or abnormally positioned teeth, often third molars. Yellow boxes mark periapical or surrounding anatomical abnormalities.

### 3.2. Methodology

**Figure 2:**
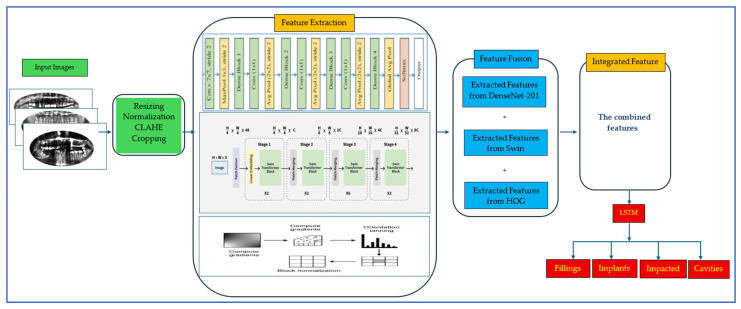
The workflow for the proposed hybrid DL framework.

To detect and diagnose dental diseases utilizing dental radiographs, a hybrid DL framework was developed. It combined HOG as handcrafted descriptors with DenseNet-201 and the Swin Transformer for transformer-based features, capturing complementary information and encompassing fine-grained low-level spatial characteristics as well as rich high-level semantic representations. Features are simultaneously extracted from HOG, DenseNet-201, and Swin and then fused to form a unified representation. The fusion process begins by integrating handcrafted HOG features with deep convolutional features from DenseNet-201. This combined representation is further enriched with high-level contextual features from the Swin Transformer, which enhances low-level texture information with deep semantic and global contextual insights. An LSTM network is utilized for classification, effectively modeling sequential dependencies within the fused features. These features are processed by a trainable fusion layer with FC layers and nonlinear activation functions, allowing the LSTM network to learn optimal fusion weights end-to-end and adjust the importance of each feature source based on its relevance. Figure 2 presents the workflow of this hybrid DL framework, and Algorithm 1 outlines the development process. The steps of the proposed framework are below:

**Phase 1 (DRAD Preprocessing)**: Firstly, we downloaded the DRAD dataset from Kaggle [13] and preprocessed the X-ray images by rescaling, normalizing, CLAHE enhancement, and cropping.

**Phase 2 (Feature Extraction)**: In the second phase, we extracted features using HOG descriptors with DenseNet-201 (DenseNet-121, MobileNet, or VGG-16) and the Swin Transformer. These features were extracted simultaneously from HOG, DenseNet-201, and Swin.

**Phase 3 (Features Fusion)**: In the third phase, we combined HOG with custom descriptors, along with DenseNet-201 (DenseNet-121, MobileNet, or VGG-16) and the Swin Transformer to leverage transformer-based features for capturing high-level image representations. Moreover, the extracted features are integrated to create a unified representation.

**Phase 4 (Pre-training CNN Techniques)**: In the fourth stage, the pre-trained CNNs (DenseNet-201, DenseNet-121, VGG-16, and MobileNet) were trained on the ImageNet dataset.

**Phase 5 (5-Fold Cross-Validation)**: In the fourth phase, the DRAD dataset was split into training and validation sets using a 5-fold cross-validation to ensure thorough model assessment. A 5-fold cross-validation technique was implemented, breaking the dataset into five parts. In every iteration, four parts were used for training, while the fifth was reserved for validation. This cycle was repeated five times, ensuring each part was tested once. This method provided a reliable evaluation of the model’s performance and minimized overfitting by enabling the model to train on diverse subsets of data.

**Phase 6 (Multi-classification)**: In the fifth phase, the unified feature vector was input into five ML methods: LSTM, random forest (RF), support vector machine (SVM), decision tree (DT), and logistic regression (LR). These models leveraged their sequential modeling capabilities to identify feature dependencies. The five ML models generated the final classification results for various dental disease categories.

**Phase 7 (Performance Evaluation)**: In the final phase, the proposed hybrid DL framework’s performance was evaluated using metrics such as accuracy, precision, recall, specificity, and F1-score.
**Algorithm 1:** The fine-tuning process of the hybrid DL Framework Steps1**Input** → *DRAD* dataset.2**Output** ← Hybrid DL Framework for Dental disease diagnosis.3**BEGIN**4      **STEP 1**: **Image Preprocessing**5  **FOR EACH** R **IN** the DRAD **DO**6              *Resize* R to 224 × 224.7              *Normalize* R’s pixel values from [0, 255] to [0, 1].8              *CLAHE*.9              *Crop R.*
10           **END FOR**
11      **STEP 2: Feature Extraction**
12            **FOR EACH** CNN **IN** [DenseNet-201, DenseNet-121, MobileNet, and VGG-16] **DO**13                  *Extract* features using HOG, CNN, and Swin.14            **END FOR**
15      **STEP 3: Features Fusion**
16           *Integrate* the extracted features from Step 2 into a unified representation.17      **STEP 4: CNN Pre-Training**
18           **FOR EACH** CNN **IN** [DenseNet-201, DenseNet-121, MobileNet, and VGG-16] **DO**19             *Pre-train* the CNN over the ImageNet dataset.20           **END FOR**
21      **STEP 5: 5 Fold Cross-Validation**
22           **FOR EACH** M **IN** [LSTM, SVM, RF, XGB, and LGB] **DO**23                      **FOR EACH i = 1 to 5**
24                           *Choose* four of the 5 folds (the training set).25                           *Use* the remaining i-th fold for the validation set.26                           *Train* the M approach on the training set and *evaluate performance on* the validation set.27                           *Calculate* the measured metrics and *compute* their average for M.28                       **END FOR**
29           **END FOR**
30      **STEP 6: Multi-Classification and Assessment**
31           **FOR EACH** M model **IN** [LSTM, RF, SVM, DT, and LR] **DO**32              *Classify* X-ray images of the testing set of the DRAD dataset.33              *Assess* the effectiveness of model M using the test set of the DRAD dataset.34           **END FOR**
35**END.**

#### 3.2.1. Data Preprocessing

Data preprocessing is essential for preparing dental X-ray images for disease detection using DL. Raw dental radiographs often exhibit inconsistent brightness, contrast, and resolution, so various enhancement and normalization methods were applied before training the ML models. To maintain uniform input dimensions across the network, all images were first resized to 224 × 224. Normalizing pixel intensities, often by scaling them to 0–1, helps stabilize the learning process and speeds up convergence. To improve diagnostic clarity, CLAHE was frequently used to enhance local contrast and emphasize fine details such as cavities, periapical infections, and bone loss. Noise reduction and denoising filters may also be applied to reduce artifacts from uneven X-ray exposure.

Additionally, the original images were cropped to create separate images for each unique class. This approach enhances dataset diversity and helps prevent overfitting. It results in multiple images derived from a single original image, particularly when the original image contains multiple labels. As a result, the training set grew from 478 to 4023, with 402 for validation and 392 for testing [13]. The distribution of the training set is presented in Table 1. Table 1 shows that each class contains a proportional number of images, maintaining a balanced dataset.

These preprocessing steps collectively ensure that DL models receive high-quality, standardized inputs that accurately capture both minor and major pathological features in dental X-rays.

#### 3.2.2. DenseNet-201 and DenseNet-121

The DenseNet family of CNNs, introduced by Huang et al. in 2017 under the title “Densely Connected Convolutional Networks” [21], presents a unique connectivity pattern. Each layer receives input from all previous layers and shares its own feature maps with all subsequent layers. DenseNet-201, with around 201 layers, is a deeper version of earlier models like DenseNet-121 and DenseNet-169.

**Figure 3:**
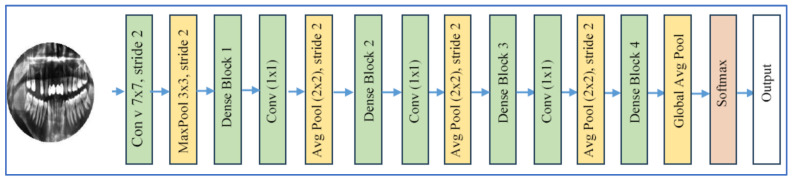
The architecture of the DenseNet-201 DL model.

Its architecture, depicted in Figure 3, consists of “dense blocks” and “transition layers.” Within dense blocks, multiple convolutional layers are interconnected, while transition layers downsample using 1 × 1 convolutions and pooling to reduce spatial resolution and feature map size. Key innovations include dense connectivity, which promotes feature sharing, mitigates the vanishing-gradient problem, and improves information transfer across layers, resulting in more efficient parameter usage [21].

In dental disease diagnosis from radiographic images, DenseNet models, including DenseNet-201 or DenseNet-121, are used to detect conditions like caries and periodontitis. For example, DenseNet-201 or DenseNet-121 have been shown to classify dental X-rays with high accuracy. DenseNet-201’s advantages in medical imaging include its strong feature extraction (capturing subtle anatomical details), effective generalization via transfer learning (pretrained on ImageNet and then fine-tuned), and efficient parameter use compared to standard deep CNNs. However, DenseNet-201 also has drawbacks: it requires substantial computational resources (memory and time), especially when trained from scratch. The concatenation of multiple feature maps can increase memory usage, and in highly imbalanced or specialized tasks (such as dental radiographs with subtle lesions), the benefits may be less than those offered by newer transformers or hybrid architectures. Additionally, the dense connectivity may complicate interpretability compared to simpler networks [21].

DenseNet-201 is based on the principle of dense connectivity, where each layer receives inputs from all preceding layers and passes its own feature maps to all subsequent layers within a dense block. Mathematically, for a DenseNet with *L* layers, the output of the lth layer (*x*_*l*_) is defined as [21]:

(1) $$x_{l}=\;H_{l}\;(x_{0},\;x_{1\;},\;\ldots ,\;x_{l-1}),$$

where $\left[x_{0},x_{1},\ldots ,x_{l-1}\right]$ denotes the concatenation of feature maps produced by all preceding layers and $H_{l}\left(\cdot \right)$ represents a composite non-linear transformation consisting of operations such as Batch Normalization (BN), Rectified Linear Unit (ReLU), 1 × 1 convolution (bottleneck layer), and 3 × 3 convolution.

Each dense block is followed by a transition layer that performs down-sampling to control feature-map growth and computational complexity:

(2) $$x_{l+1}=Pooling\;(W_{l\;},*\;x_{l}+\;b_{l}),$$

where $b_{l}\;$ and $W_{l}\;$ are the convolutional biases and weights, and ∗  denotes convolution.

The growth rate (k) determines the number of feature maps added by each layer, maintaining parameter efficiency. In DenseNet-201, a relatively small growth rate enables a deeper architecture (201 layers) without excessive parameter growth. The final feature representation is passed through a global average pooling layer, followed by a fully connected (FC) layer and softmax activation for classification:

(3) $$\hat{y}=softmax(W_{fc}.x_{final}+b_{fc}).$$

#### 3.2.3. VGG-16

**Figure 4:**
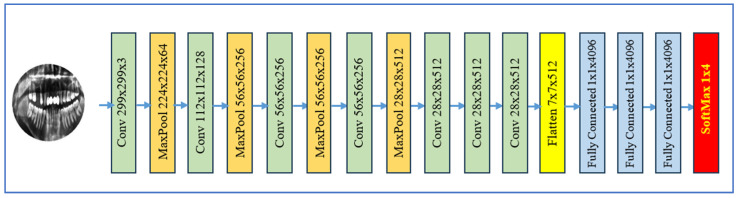
The architecture of the VGG-16 DL model.

The VGG-16 is a deep CNN with 16 weight layers. Developed by the Visual Geometry Group at the University of Oxford, it was introduced in 2014 by Karen Simonyan and Andrew Zisserman [22]. This network is characterized by its straightforward, uniform design, which emphasizes depth through the use of small convolutional kernels. As depicted in Figure 4, the network processes a 224 × 224 RGB image through five consecutive convolutional blocks. Each block comprises multiple 2-D convolutional layers featuring 3 × 3 kernels, a stride of 1, and “same” padding, followed by a 2 × 2 max-pooling layer with a stride of 2, halving the spatial resolution. The depth of the convolutional layers increases across the blocks, progressing from 64 to 128 to 256 to 512 to 512 filters, thereby enhancing feature extraction at deeper levels. VGG-16 achieved strong performance in the ImageNet Large-Scale Visual Recognition Challenge 2014, contributing to the popularity of deep, uniform CNN architectures.

Its simplicity and effective transfer learning on limited medical data are notable strengths, alongside its high feature representation enabled by depth and small kernels. However, it has a high parameter count (~138 M), requires significant memory/storage, and offers slower inference compared to modern lightweight models. VGG-16 serves as a core backbone for extracting features in many medical and dental DL applications, especially from radiographs or intra-oral images. In dental disease detection, it is used to identify conditions like caries, periapical lesions, and periodontal bone loss from X-rays or clinical images. VGG-16 was adapted for detecting tooth decay and abnormalities in panoramic and periapical radiographs, proving its effectiveness in pathological recognition [22].

#### 3.2.4. MobileNet

**Figure 5:**
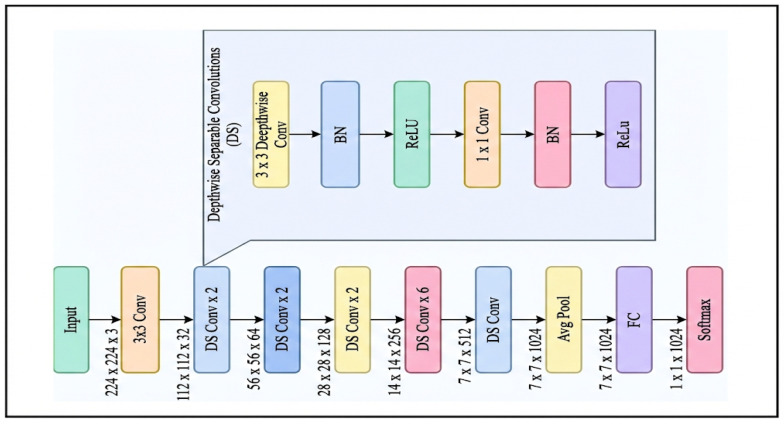
The architecture of the MobileNet DL model.

In 2017, researchers at Google Brain introduced MobileNet, aiming to make CNNs more efficient for mobile and embedded vision systems [23]. MobileNet’s architecture, as shown in Figure 5, focuses on efficiency by utilizing depthwise separable convolutions. This approach divides a standard convolution into two steps: a depthwise operation, which applies a (k × k) filter to each input channel, followed by a pointwise (1 × 1) convolution to mix the channels. The series evolved with MobileNet-V2 in 2018, introducing inverted residuals and linear bottlenecks, and later with MobileNet-V3 in 2019, which used Neural Architecture Search (NAS) and squeeze-and-excite modules to improve latency-accuracy balance.

The main benefits include a small memory footprint, rapid inference, and strong performance when pretrained and fine-tuned, making it suitable for high-resolution or real-time applications on limited hardware. However, it has limitations, including reduced representational capacity compared to heavier models, sensitivity to aggressive compression, and potential accuracy loss on detailed or imbalanced medical tasks [23].

In dental disease detection—covering issues such as caries, periodontal disease, periapical lesions, and oral mucosal conditions—MobileNet serves as an essential lightweight feature extractor for portable or chair-side deep learning models, enabling deployment on intraoral imaging devices or low-compute clinical systems. Its efficiency enables the processing of dental radiographs and oral photographs and their integration into CAD systems without significant delay, which is particularly beneficial for screening for dental caries and periodontal disease in resource-limited environments. Recent research shows that MobileNet backbones offer competitive diagnostic capability for classifying and localizing dental diseases when paired with attention modules or multi-task heads, while also supporting deployment on edge hardware in dental clinics [23].

#### 3.2.5. Swin Transformer

The Swin Transformer, also known as the Shifted-Window Transformer, is an advanced vision transformer architecture that acts as an effective image encoder in DL models. It computes self-attention within distinct local windows and periodically shifts them to facilitate cross-region interactions. It was introduced in 2021 by Microsoft Research in the paper titled “Swin Transformer: Hierarchical Vision Transformer using Shifted Windows,” presented at the ICCV 2021 conference [24].

The Swin architecture is a layered Vision Transformer (ViT) that incorporates local and cross-region interactions via a unique windowing approach. It starts with a Patch Embedding layer, using a strided convolution to transform an input scan $I\;\in R^{H\;*\;W*\;3}$ into a sequence of non-overlapping patch tokens $X_{0}\in \;R^{N\;*\;C}\;$, where (N = $\frac{HW}{P^{2}}$) and (C) is the hidden dimension. The network builds a multi-scale hierarchy by stacking Swin Transformer Blocks in stages, progressively reducing spatial resolution via Patch Merging. This process concatenates features from (2 × 2) neighboring tokens and projects them with a learned linear layer to increase channel capacity while reducing sequence length.

Unlike global self-attention in ViTs, each attention layer in Window Multi-Head Self-Attention (W-MSA) limits computation to fixed-size windows (M × M), reducing complexity from quadratic to linear with respect to the scan size. The model alternates with Shifted Window Multi-Head Self-Attention (SW-MSA), where feature maps are cyclically shifted by (M/2, M/2) before window partitioning and masked attention is applied, allowing cross-window connections without the cost of global attention. Each block uses a pre-LayerNorm design, including a residual attention sublayer followed by a two-layer MLP with GELU nonlinearity, maintaining standard Transformer learning dynamics and supporting large-scale dense vision tasks such as classification, detection, and segmentation [24].

**Figure 6:**
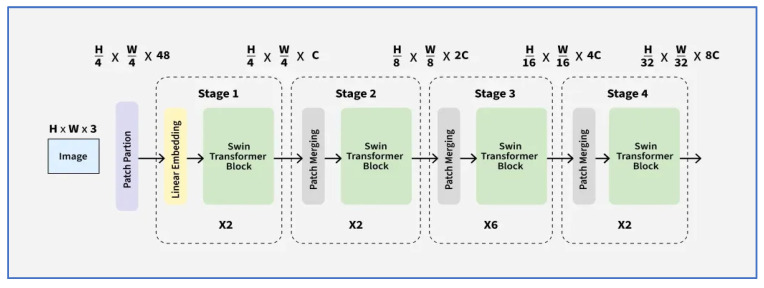
The Swin Transformer’s architecture.

The hierarchical feature maps maintain spatial locality akin to CNNs, enhancing transfer learning capabilities. However, challenges include increased architectural complexity from the shifted-window partitioning, dependence on window-size tuning, and higher inference latency compared to lightweight CNNs. In the context of dental disease detection using DL, Swin is crucial for identifying detailed structural and semantic patterns in intraoral and radiographic images. It improves the identification of subtle infection markers, tooth decay boundaries, periodontal bone loss, and lesion textures. Several studies have shown that Swin-based backbones boost classification and segmentation accuracy for detecting dental caries, periapical lesions, and periodontal disease when incorporated into downstream frameworks [24]. Figure 6 depicts the Swin’s architecture.

#### 3.2.6. ViT

**Figure 7:**
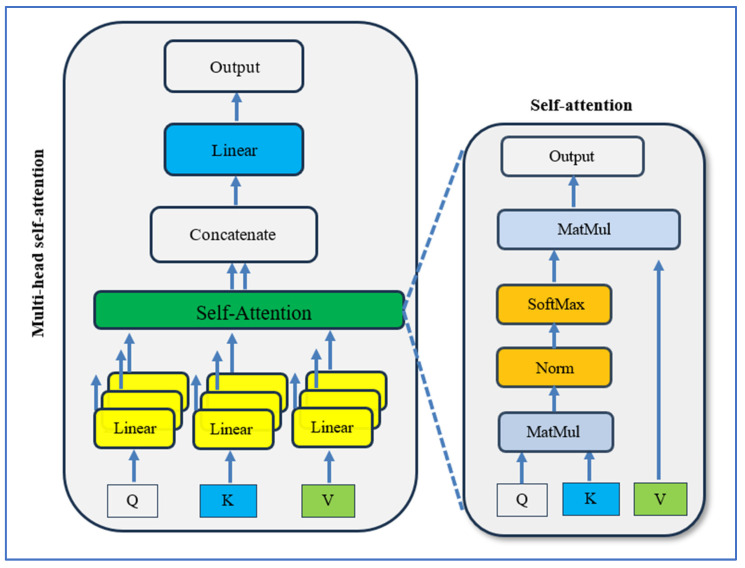
The process of the multi-head self-attention.

**Figure 8:**
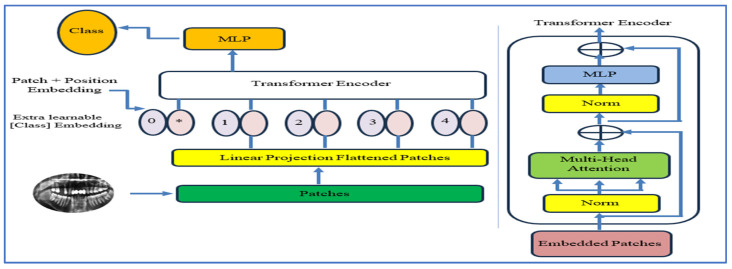
The ViT Transformer’s architecture and * denotes to special learnable classification token.

The ViT, introduced by Alexey Dosovitskiy and his team at Google Research in 2020, demonstrated that a pure Transformer model could achieve top-tier image recognition performance when trained on large datasets and deployed on powerful hardware [25]. Unlike convolutional networks, ViT divides an input image into fixed, non-overlapping patches, flattens them, and learns an embedding vector for each patch. These vectors are combined with positional encodings and processed through stacked multi-head self-attention (MSA) and feed-forward MLP layers, using pre-LayerNorm and residual connections. This design provides a global receptive field from the first layer, allowing for effective modeling of long-range relationships. Figure 7 and Figure 8 show the self-attention process and the ViT architecture, respectively.

Notable benefits include scalability, efficient parallel training, and the ability to capture distributed visual dependencies, leading to strong transfer performance on detailed tasks. However, the model has quadratic attention complexity $𝒪(N^{2})$ concerning the number of patches (*N*), demands high memory and computational power at large resolutions, and has a weaker inductive bias compared to CNNs, making it data-demanding and sensitive to patch and positional encoding choices. In dental and oral imaging, Transformers have become increasingly helpful in identifying disorders characterized by spatially distributed or subtle structural irregularities. Global attention helps relate patterns across teeth and surrounding structures, which is particularly helpful in detecting conditions like caries, periodontal bone loss, and other oral abnormalities where pathology may spread across multiple areas rather than being localized. According to Transformers for Medical Image Analysis, these benefits extend to high-resolution X-ray technologies when complexity is carefully managed [25].

#### 3.2.7. HOG

**Figure 9:**
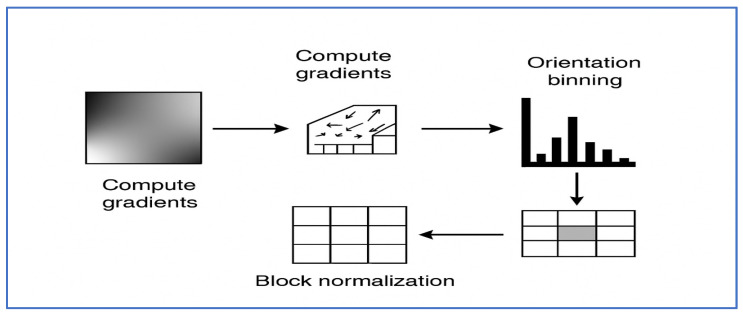
The HOG algorithm’s steps.

The HOG feature descriptor was officially introduced in 2005 by Navneet Dalal and Bill Triggs through a paper titled “Histograms of Oriented Gradients for Human Detection” [26] for pedestrian detection. However, it builds on earlier concepts of gradient-orientation histograms from the 1980s. Architecturally, as illustrated in Figure 9 and Algorithm 2, HOG functions by segmenting an image into small spatial “cells” (e.g., 8 × 8 pixels), calculating the gradient magnitude and direction for each pixel, creating a local histogram of gradient orientations for each cell (usually with 9 bins), then combining adjacent cells into overlapping “blocks” for contrast normalization, and finally assembling all normalized block histograms into a comprehensive feature vector for classification.

In dental disease diagnosis, HOG can be used as a handcrafted descriptor to identify low-level structural and edge-based patterns in dental radiographs (such as cavity boundaries, root edges, or bone loss contours), which enhance more advanced DL features. By integrating HOG into a hybrid framework, its interpretability and edge sensitivity can be leveraged. The benefits of HOG include its relatively low computational cost (compared to many deep networks), its interpretability (due to its basis in edge/gradient distributions), and its effectiveness when data is limited or features are straightforward. However, in contemporary dental imaging, its limitations are notable: HOG is unable to capture high-level semantic features (like subtle tissue textures, complex pathology patterns, or contextual information) and is sensitive to scale and rotation unless specifically engineered. In many large-scale or complex tasks, it is outperformed by deep convolutional or transformer-based feature extractors.

In summary, HOG is valuable as a complementary or fallback feature technique in dental image analysis frameworks, especially when interpretability and simplicity are essential. However, it is unlikely to achieve the performance of modern deep learning methods on its own.
**Algorithm 2:** The HOG technique1**Input** → *Image.*2**Output** → Feature vector.3**BEGIN**4     **STEP 1**: **Image Preprocessing**5   convert_to_grayscale (Image) ⟶ Image6            gamma_normalize (Image) ⟶ Image7     **STEP 2: Gradient Computation**
8            convolve (Image, [−1, 0, 1]) → Gx.9            convolve (Image, ${\left[-1,\;0,\;1\right]}^{T}$) →*Gy.*10          **FOR EACH pixel** (i, j) **DO**11               sqrt (${Gx[i,\;j]}^{2}$ + ${Gy[i,\;j]}^{2}$)  → M[i, j].12               arctan2 (Gy[i, j], Gx[i, j]) → θ[i, j].13            **END FOR**
14     **STEP 3: Divide the image into cells**
15            split_image_into_cells (Image, cell_size = 8 × 8) → cells.16     **STEP 4: Orientation binning**
17            **FOR EACH** cell **DO**18                   9 bins from 0° to 180° → histogram.19                   **FOR EACH** pixel in cell **DO**20                        corresponding_bin (θ) → bin21                        histogram [bin] + = M [pixel]22                      **END FOR**
23                 cell_histograms.append (histogram)24            **END FOR**
25     **STEP 5: Group Cells into Blocks**
26           group_cells_into_overlapping_blocks (cell_histograms, block_size = 2 × 2) → blocks.27     **STEP 6: Block Normalization**
28          **FOR EACH** block **DO**29                concatenate_histograms (block) → h.30                normalize (h, L2_norm or L1_norm) → h_norm.31                normalized_block_histograms.append (h_norm).32           **END FOR**
33     **STEP 7: Feature vector construction**
34           concatenate_all (normalized_block_histograms) → feature_vector.35**END.**

The HOG represents the local shape of an image by capturing the distribution of intensity gradients or edge orientations. For a grayscale image I(x, y), the initial step involves calculating the image gradients in both the horizontal and vertical directions using discrete derivative filters, such as:

Gx = I(x + 1, y) − I(x − 1, y), Gy = I(x, y + 1) − I(x, y − 1).(4)

The magnitude and orientation of the gradient at each pixel are then obtained as:

(5) $$M\left(x,y\right)=\;\sqrt{G_{x}^{2}+G_{y}^{2}\;},\;\theta \;\left(x,y\right)=\;\tan^{-1}\left(\frac{G_{y}}{G_{x}}\right).$$

The image is divided into small, spatially connected regions called cells (e.g., 8×8 pixels). Within each cell, a histogram of gradient orientations is computed, typically using 9 bins covering 0–180° (unsigned) or 0–360° (signed) angles. Each pixel contributes to its orientation bin proportionally to its magnitude M(x,y).

To achieve illumination and contrast invariance, histograms from adjacent cells are grouped into blocks (e.g., 2×2 cells) and normalized using one of several normalization schemes, such as L2-norm:

(6) $$v_{norm}=\;\frac{v}{\sqrt{{\left\|v\right\|}^{2}+\epsilon^{2}}},$$

where v is the concatenated histogram vector from the block and ϵ  is a small constant to avoid division by zero. Finally, all normalized block histograms are concatenated into a single high-dimensional feature vector representing the entire image or region of interest:

(7) $$HOG\;(I)\;[v_{1},\;v_{2},\ldots ,v_{n}].$$

This vector serves as the input to classifiers such as SVMs or, in hybrid deep learning systems, to dense or convolutional layers for further learning.

#### 3.2.8. LSTM

**Figure 10:**
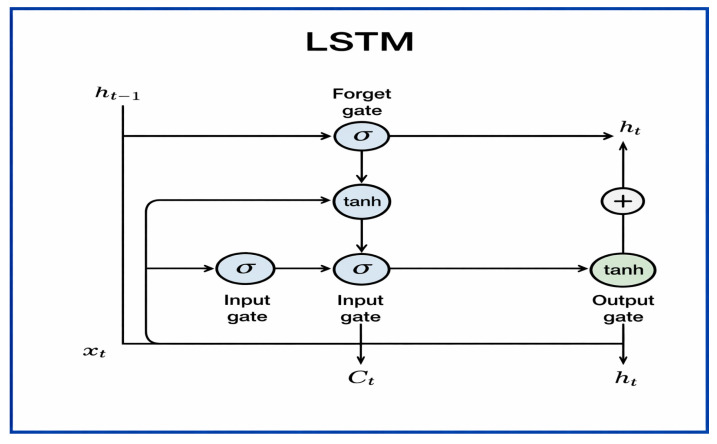
The LSTM model’s architecture.

LSTM is a unique kind of recurrent neural network (RNN) created by Sepp Hochreiter and Jürgen Schmidhuber in 1997 [27]. It was designed to solve the vanishing and exploding gradient issues that hindered traditional RNNs from learning long-term patterns in sequential data. The LSTM structure depicted in Figure 10 includes memory cells with three primary gates—input, forget, and output—that manage the flow of information by determining what to retain, update, or discard over time. This gating system allows LSTMs to capture both short- and long-term dependencies, making them highly effective for modeling sequential data.

In diagnosing dental diseases, LSTMs have been used to analyse sequential features from medical images and combined feature representations. When integrated with CNNs or manual descriptors, LSTMs can detect patterns across multiple feature maps or slices, thereby improving the accuracy of identifying dental caries, periapical lesions, and periodontal diseases in radiographic images.

LSTMs excel at maintaining long-term contextual information, adapting to different data types (temporal, spatial, or hybrid), and handling complex feature dependencies. However, they also have drawbacks, such as higher computational requirements, longer training times, and a tendency to overfit on small or unbalanced datasets. Despite these challenges, LSTMs are essential in deep learning for medical imaging, offering a robust method for sequential feature learning and diagnostic decision-making in dentistry.

Each gate (input, forget, and output) employs a sigmoid activation function to determine how much information to keep or discard. At the same time, the cell state uses a tanh activation function for updates. At time step t, for an input vector $x_{t}$ and the previous hidden state $h_{t-1}$, the LSTM equations are:

(8) $$f_{t}=\sigma (W_{f}[h_{t-1},x_{t}]+b_{f})\;\;\;\;\;\;\;\;\;\;\;\;\;\;\;\;\;\;\;\;(\mathrm{Forget}\;\mathrm{gate}),$$

(9) $$i_{t}=\sigma (W_{i}[h_{t-1},x_{t}]+b_{i})\;\;\;\;\;\;\;\;\;\;\;\;\;\;\;\;\;\;\;\;\;\;(\mathrm{Input}\;\mathrm{gate}),$$

(10) $$\tilde{C_{t}}=\tanh(W_{C}[h_{t-1},x_{t}]+b_{C})\;\;\;(\mathrm{Candidate}\;\mathrm{cell}\;\mathrm{gate}),$$

(11) $$C_{t}=f_{t}*C_{t-1}+i_{t}*\;{\tilde{C}}_{t}\;\;\;\;\;\;\;\;\;\;\;\;\;\;\;\;\;\;\;\;\;\;\;(\mathrm{Updated}\;\mathrm{cell}\;\mathrm{gate}),$$

(12) $$o_{t}=\sigma (W_{o}[h_{t-1},x_{t}]+b_{o})\;\;\;\;\;\;\;\;\;\;\;\;\;\;\;\;\;\;\;(\mathrm{Output}\;\mathrm{gate}),$$

(13) $$h_{t}=o_{t}*\tanh(C_{t})\;\;\;\;\;\;\;\;\;\;\;\;\;\;\;\;\;\;\;\;\;\;\;\;\;(\mathrm{New}\;\mathrm{hidden}\;\mathrm{state}),$$

where $f_{t}$, $i_{t}$, and $o_{t}$ are gate activations controlling information retention, update, and output, respectively. $C_{t}\;$ is the cell state, acting as memory across time steps. $h_{t}\;$ is the hidden state, representing the output at each step. W and b denote weight matrices and bias terms for each gate. σ is the sigmoid activation function, and tanh is the hyperbolic tangent function. This mathematical framework allows LSTM networks to maintain long-term dependencies by managing gradient flow during backpropagation, thereby reducing the vanishing gradient problem often encountered in regular RNNs.

#### 3.2.9. Four ML Techniques

In this research, we used four additional ML approaches to assess the performance of the LSTM model: SVM [28], RF [29], DT [30], and LR [31]. A comparative analysis of these models is shown in Table 2.

#### 3.2.10. Evaluated Performance Metrics

The performance of the proposed framework using the four DL and five ML models was assessed using the equations outlined in Equations (14)–(18)

(14) $$Accuracy=\frac{\left(TP+TN\right)}{\left(TP+FP+TN+FN\right)},$$

(15) $$Precision=\frac{TP}{\left(TP+FP\right)},$$

(16) $$Sensivity=\frac{TP}{\left(TP+FN\right)},$$

(17) $$Specifity=\frac{TN}{\left(TN+FP\right)},$$

(18) $$F1-score=\frac{2\times Precision\times Recall}{Precision+Recall}.$$

True Positive (*TP*) represents cases in which the model correctly predicts a positive outcome and the ground-truth labels confirm the presence of the condition. True Negative (*TN*) corresponds to instances in which the model correctly identifies negative cases, in agreement with the dataset annotations. A False Positive (*FP*) occurs when the model incorrectly classifies a case as positive, even though the actual condition is negative (e.g., predicting a tumor when none exists). Conversely, a False Negative (*FN*) occurs when the model fails to detect a positive case and incorrectly predicts a negative outcome, even though the condition is truly present (such as an undetected tumor).

#### 3.2.11. Evaluation Environment

In this paper, we performed four experiments to evaluate the performance of the proposed model on the DRAD dataset. The experiments were conducted on an Intel i9-class processor, 16 GB of RAM, fast SSD storage, and efficient cooling. The used hyperparameters are shown in Table 3.

Table 3 outlines the setup applied in the experiment. The input images were standardized to a spatial resolution of 224 × 224 pixels with 3 RGB channels, ensuring compatibility with the static backbone. The pipeline incorporated normalization, resizing, and cropping as preprocessing steps to ensure uniformity across samples. For spatial feature aggregation, the model used Adaptive Average Pooling (AAP), which dynamically adjusted pooling kernels to produce fixed-length outputs regardless of input differences. Since the backbone was static, training elements such as the optimizer and loss function were not used, and no gradient updates or backpropagation occurred. The framework operated solely in inference mode, focusing on extracting deep descriptors. The final output of the pooling layer was a 512-dimensional feature vector, serving as a compact embedding for subsequent machine learning classifiers and analysis tasks.

## 4. Results and Interpretation

The four experiments were primarily designed to detect dental diseases, with the objectives of improving patient outcomes, streamlining diagnostic workflows, and significantly reducing the time and financial burden on patients. We used the DRAD dataset, which was divided into training and testing sets. The training set contains 4023 images, and the test set contains 392 images. We trained DenseNet-201, DenseNet-121, VGG-16, and MobileNet over the ImageNet dataset.

In the first experiment, we used four DL models and two transformers (Swin and ViT) to extract deep features and capture high-level image representations. Each model was used independently, with five ML models serving as classifiers. In the second experiment, we combined HOG with DL models and two transformers to extract handcrafted features for effective identification of structural patterns. Meanwhile, the DL models and transformers continued to extract deep features, and the ML models acted as classifiers. In the third experiment, we used the two transformers (Swin and ViT) alongside the DL models for feature extraction. Finally, in the fourth experiment, we combined DL models and two transformers for deep feature extraction, with HOG for handcrafted feature detection, again employing ML models as classifiers.

The four experiments employed a 5-fold cross-validation strategy to ensure robust, unbiased evaluation of model generalization. In this approach, the training dataset was partitioned into five equally sized subsets. During each iteration, one subset was held out for validation. In contrast, the remaining four subsets were used to train the model, allowing the model parameters to be updated independently in each cycle. This procedure was repeated five times so that each subset served as the validation set exactly once. The final performance of each model was obtained by averaging the results across all folds, thereby reducing variance and mitigating the risk of overfitting. At the end of the experimental process, the evaluation metrics defined in Equations (14)–(18) were computed and averaged for each of the five machine learning techniques to provide a comprehensive assessment of their predictive performance.

In the first experiment, we used four DL models, including Swin and ViT transformers, to extract deep features and capture high-level image representations. Each model was used independently, with five ML models serving as classifiers. The results of the five-fold cross-validation for the five ML models, along with their corresponding evaluation metrics, are presented in Table 4, Table 5, Table 6, Table 7, Table 8 and Table 9.

**Figure 11:**
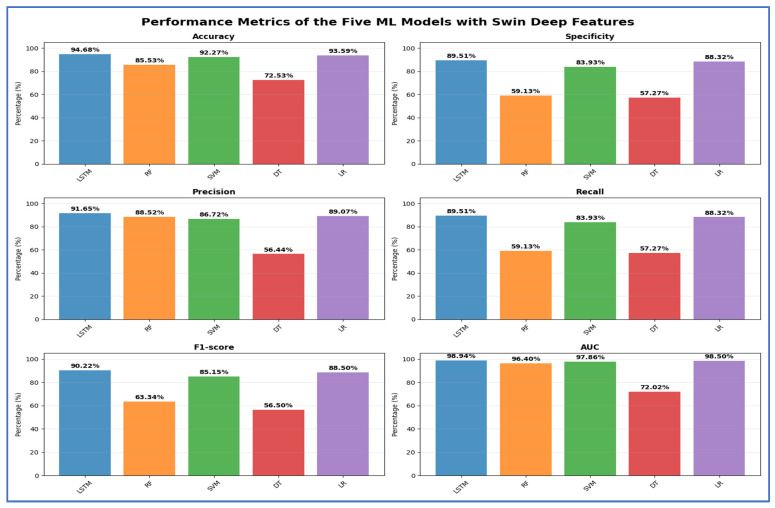
The performance metrics of the five ML approaches with Swin Transformer deep features.

Table 4 and Figure 11 present the cross-validation metrics for the five ML models using the Swin Transformer to extract deep features. The Swin–LSTM pipeline achieved the best overall results, with 94.68% accuracy, 89.51% specificity, 91.65% precision, 89.51% recall, 90.22% F1-score, and an AUC of 98.94%, demonstrating strong discriminative capabilities and balanced sensitivity and precision. Traditional ML classifiers implemented with established libraries yielded varied results: the RF model achieved 85.53% accuracy and 96.40% AUC, but its low recall (59.13%) hindered its ability to identify positive cases effectively, despite a high precision of 88.52%. The SVM classifier performed well, achieving 92.27% accuracy and an AUC of 97.86%, while maintaining a high F1-score of 85.15%. At the same time, the DT showed the lowest performance, with an accuracy of 72.53% and an AUC of 72.02%, indicating limited generalization and unstable predictive reliability. LR maintained competitive results, with 93.59% accuracy and an AUC of 98.50%, demonstrating that linear decision boundaries can be effective when combined with strong deep representations. Networks inspired by depthwise efficiency for feature extraction and recurrent gating for temporal aggregation have been recognized as complementary in detailed medical imaging tasks, such as oral radiographic analysis.

**Table 4:** The mean cross-validation scores for the five ML models using the Swin Transformer.

Deep Features	ML	Accuracy (%)	Specificity (%)	Precision (%)	Recall (%)	F1-Score (%)	AUC (%)
Swin	LSTM	94.68	89.51	91.65	89.51	90.22	98.94
	RF	85.53	59.13	88.52	59.13	63.34	96.40
	SVM	92.27	83.93	86.72	83.93	85.15	97.86
	DT	72.53	57.27	56.44	57.27	56.50	72.02
	LR	93.59	88.32	89.07	88.32	88.50	98.50

**Figure 12:**
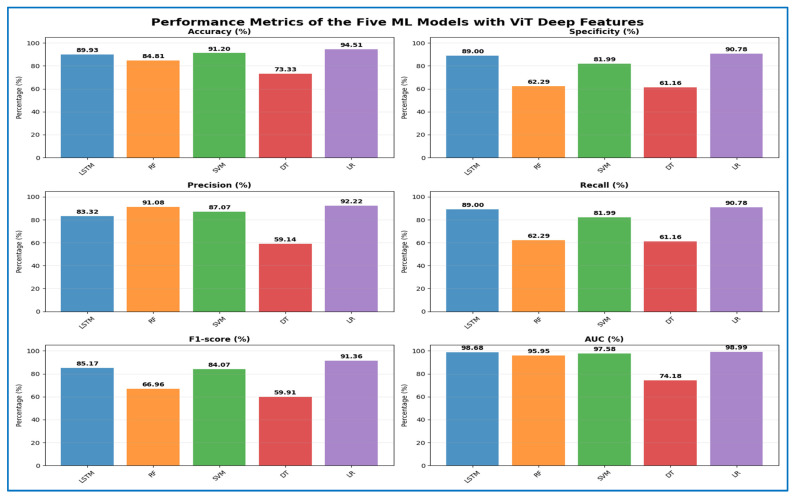
The performance metrics of the five ML approaches with ViT Transformer deep features.

Table 5 and Figure 12 present the cross-validation metrics for five ML models using the ViT transformer to extract deep features. The LR classifier outperformed others, achieving a 94.51% accuracy, 90.78% specificity, 92.22% precision, 90.78% recall, and an F1-score of 91.36%, along with an impressive 98.99% AUC, indicating well-balanced discrimination and high reliability. The SVM classifier was a close second, with 91.20% accuracy, 81.99% recall, 87.07% precision, and 97.58% AUC, demonstrating strong performance on positive anomalies. The RF produced moderate results, achieving 84.81% accuracy with lower recall and specificity (62.29%), while maintaining high precision (91.08%), suggesting a conservative predictive tendency. DT had the weakest performance, with 73.33% accuracy, 59.91% F1-score, and only 74.18% AUC, indicating limited ability to separate complex features. The LSTM classifier achieved 89.93% accuracy, 89% recall, 83.32% precision, and the highest anomaly sensitivity among non-global models, with an excellent 98.68% AUC, showing that sequential modeling enhanced the transformer’s features. Overall, depthwise-factorized linear models and kernel-based separators benefited most from the globally encoded transformer map, while shallow tree-based classifiers struggled with the dense.

**Table 5:** The mean cross-validation scores for the five ML models using the ViT transformer.

	ML	Accuracy (%)	Specificity (%)	Precision (%)	Recall (%)	F1-Score (%)	AUC (%)
ViT	LSTM	89.93	89.00	83.32	89.00	85.17	98.68
	RF	84.81	62.29	91.08	62.29	66.96	95.95
	SVM	91.20	81.99	87.07	81.99	84.07	97.58
	DT	73.33	61.16	59.14	61.16	59.91	74.18
	LR	94.51	90.78	92.22	90.78	91.36	98.99

**Figure 13:**
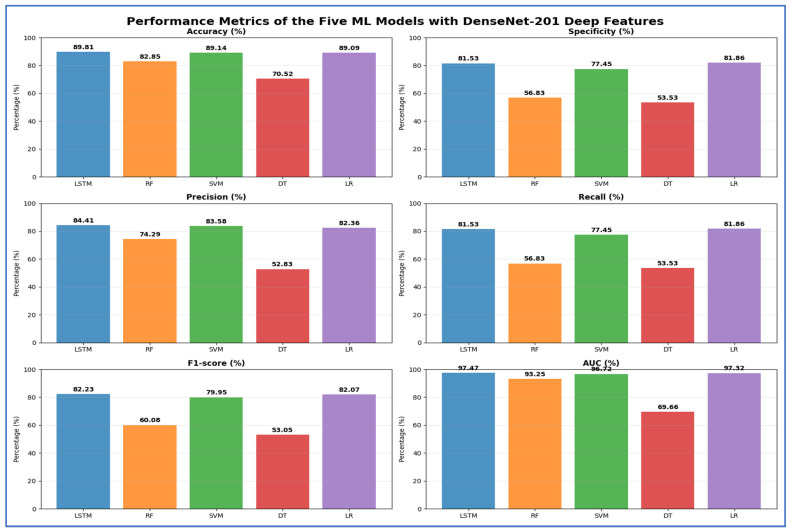
The performance metrics of the five ML approaches with DenseNet-201 DL model deep features.

In Table 6 and Figure 13, the LSTM model achieved the highest accuracy at 89.81%, with a specificity of 81.53%, precision of 84.41%, recall of 81.53%, F1-score of 82.23%, and an AUC of 97.47%. The SVM model showed similar results, with an accuracy of 89.14%, specificity of 77.45%, precision of 83.58%, recall of 77.45%, F1-score of 79.95%, and an AUC of 96.72%. LR also performed well, achieving 89.09% accuracy, 81.86% specificity, 82.36% precision, 81.86% recall, 82.07% F1-score, and 97.32% AUC. The RF model had moderate results, with an accuracy of 82.85%, but lower specificity (56.83%), recall (56.83%), and F1-score (60.08%), along with an AUC of 93.25%. The DT model had the lowest performance, with an accuracy of 70.52%, specificity of 53.53%, precision of 52.83%, recall of 53.53%, F1-score of 53.05%, and an AUC of 69.66%. Overall, LSTM and LR emerged as the top-performing models, while DT showed the least effective.

**Table 6:** The mean cross-validation scores for the five ML models using DenseNet-201.

Deep Features	ML	Accuracy (%)	Specificity (%)	Precision (%)	Recall (%)	F1-Score (%)	AUC (%)
DenseNet-201	LSTM	89.81	81.53	84.41	81.53	82.23	97.47
	RF	82.85	56.83	74.29	56.83	60.08	93.25
	SVM	89.14	77.45	83.58	77.45	79.95	96.72
	DT	70.52	53.53	52.83	53.53	53.05	69.66
	LR	89.09	81.86	82.36	81.86	82.07	97.32

**Figure 14:**
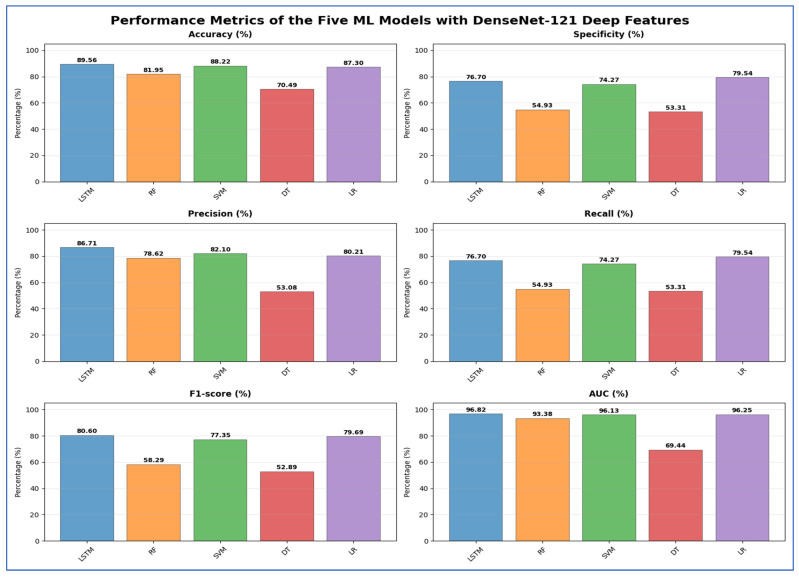
The performance metrics of the five ML approaches with DenseNet-121 DL model deep features.

Table 7 and Figure 14 displayed the results of the five ML models utilizing DenseNet-121 deep features. The LSTM model achieved the highest accuracy at 89.56% and a specificity of 76.70%. Its precision was 86.71%, yielding a recall of 76.70%, an F1-score of 80.60%, and an AUC of 96.82%. The SVM model showed slightly lower accuracy (88.22%), with specificity of 74.27%, precision of 82.10%, recall of 74.27%, F1-score of 77.35%, and AUC of 96.13%. LR achieved an accuracy of 87.30%, with a higher specificity of 79.54%, precision of 80.21%, recall of 79.54%, F1-score of 79.69%, and AUC of 96.25%. RF showed moderate performance, with an accuracy of 81.95%; however, its specificity and recall were notably lower at 54.93%, while precision was 78.62% and AUC was 93.38%. DT had the weakest overall performance, with an accuracy of 70.49%, a specificity of 53.31%, a precision of 53.08%, a recall of 53.31%, an F1-score of 52.89%, and an AUC of 69.44%. Overall, LSTM surpassed the other models in terms of accuracy and AUC, while the DT had the lowest performance across most metrics.

**Table 7:** The mean cross-validation scores for the five ML models using DenseNet-121.

Deep Features	ML	Accuracy (%)	Specificity (%)	Precision (%)	Recall (%)	F1-Score (%)	AUC (%)
DenseNet-121	LSTM	89.56	76.70	86.71	76.70	80.60	96.82
	RF	81.95	54.93	78.62	54.93	58.29	93.38
	SVM	88.22	74.27	82.10	74.27	77.35	96.13
	DT	70.49	53.31	53.08	53.31	52.89	69.44
	LR	87.30	79.54	80.21	79.54	79.69	96.25

**Figure 15:**
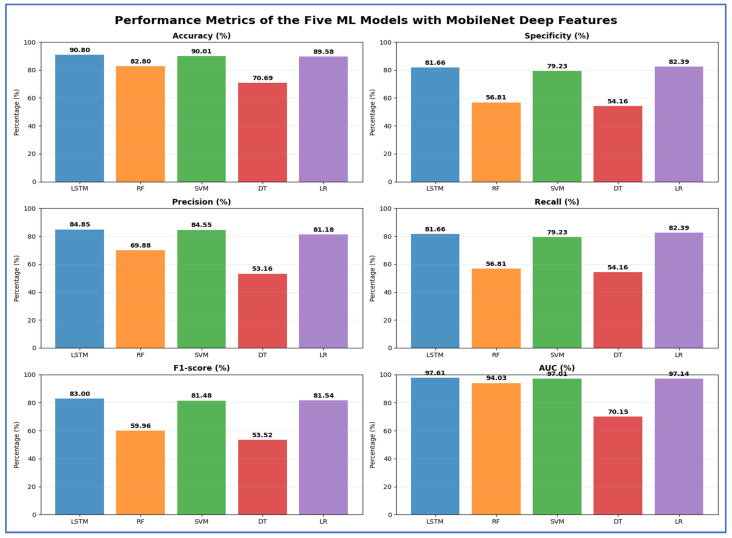
The performance metrics of the five ML approaches with MobileNet DL model deep features.

Table 8 and Figure 15 present the evaluation of the five ML models applied to deep features extracted from MobileNet. Overall, LSTM achieved the highest accuracy of 90.80% and a strong AUC of 97.61%, indicating effective discrimination between classes. SVM closely matched LSTM, achieving 90.01% accuracy and 97.01% AUC, demonstrating robust classification performance. RF achieved moderate accuracy (82.80%), but its specificity and recall were relatively low (56.81%), suggesting it struggled to correctly identify negative cases. DT exhibited the lowest overall performance, with an accuracy of 70.69% and an AUC of 70.15%, reflecting limited predictive capability. LR achieved competitive results, with 89.58% accuracy and 82.39% specificity, suggesting it effectively balanced the detection of positive and negative cases. In summary, the results demonstrated that LSTM and SVM were the most effective models for the given deep features. In contrast, RF and DT underperformed, and LR provided a reliable alternative with slightly lower accuracy but consistent specificity.

**Table 8:** The mean cross-validation scores for the five ML models using MobileNet.

Deep Features	ML	Accuracy (%)	Specificity (%)	Precision (%)	Recall (%)	F1-Score (%)	AUC (%)
MobileNet	LSTM	90.80	81.66	84.85	81.66	83.00	97.61
	RF	82.80	56.81	69.88	56.81	59.96	94.03
	SVM	90.01	79.23	84.55	79.23	81.48	97.01
	DT	70.69	54.16	53.16	54.16	53.52	70.15
	LR	89.58	82.39	81.18	82.39	81.54	97.14

**Figure 16:**
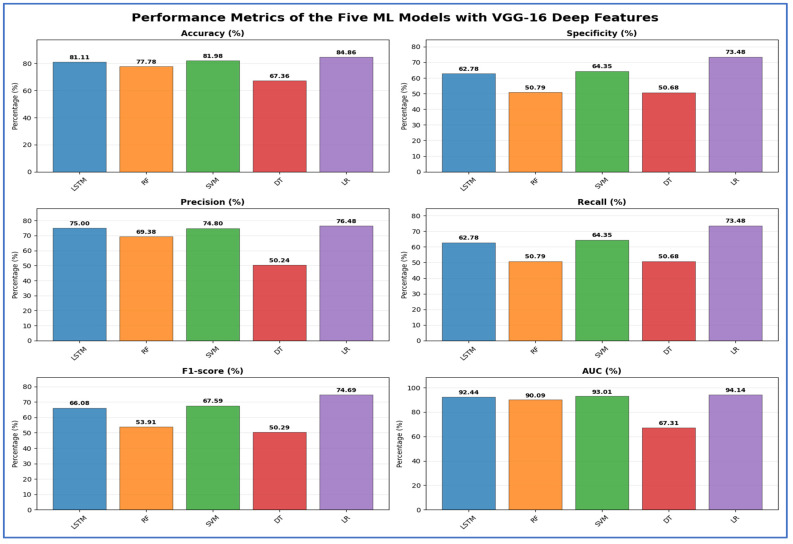
The performance metrics of the five ML approaches with VGG-16 DL model deep features.

Table 9 and Figure 16 present the results for models using VGG-16 as a deep feature extractor with the five ML classifiers. LR achieved the highest peak accuracy of 84.86%, while SVM and LSTM achieved 81.98% and 81.11%, respectively. The DT model had the lowest accuracy (67.36%) and AUC (67.31%), indicating poor discriminative capability. RF showed a moderate overall performance with 77.78% accuracy, but both recall and specificity dropped to 50.79%, highlighting challenges in reliably identifying both negative and positive classes. LR also achieved the highest specificity (73.48%) and the strongest AUC (94.14%) among the classifiers, indicating better generalization with fewer false positives. Although LSTM and SVM had similar accuracy, SVM had slightly higher recall (64.35%) and F1-score (67.59%) than LSTM (66.08%), making it the second-most balanced model after LR.

**Table 9:** The mean cross-validation scores for the five ML models using VGG-16.

Deep Features	ML	Accuracy (%)	Specificity (%)	Precision (%)	Recall (%)	F1-Score (%)	AUC (%)
VGG-16	LSTM	81.11	62.78	75.00	62.78	66.08	92.44
	RF	77.78	50.79	69.38	50.79	53.91	90.09
	SVM	81.98	64.35	74.80	64.35	67.59	93.01
	DT	67.36	50.68	50.24	50.68	50.29	67.31
	LR	84.86	73.48	76.48	73.48	74.69	94.14

**Figure 17:**
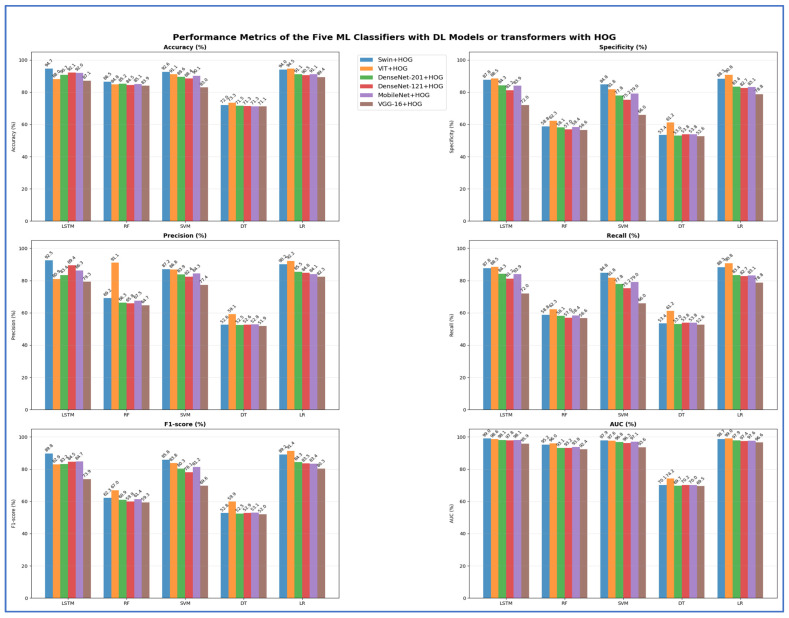
The performance metrics of the five ML approaches using DL models or Transformers with HOG.

In the second experiment, we integrated HOG with DL models and two transformers to extract handcrafted features for efficiently identifying structural patterns. Meanwhile, the DL models and transformers continued to extract deep features, while ML models served as classifiers. Table 10 and Figure 17 display the results of the five-fold cross-validation for the five ML models, along with their evaluation metrics.

The Swin + HOG with LSTM model achieved the highest training accuracy at 94.68%, followed closely by ViT + HOG with LR at 94.51% and Swin + HOG with LR at 94.01%. Transformer-based backbones, particularly Swin and ViT, consistently achieved high accuracy across classifier types, with both LSTM and linear classifiers performing exceptionally well. Among CNN backbones, DenseNet-201 and DenseNet-121 achieved strong peak accuracies (90.68% and 92.12%, respectively), while MobileNet demonstrated high efficiency, reaching 92.02%. However, CNN backbones experienced notable performance declines when paired with non-neural or tree-based classifiers. The lowest accuracy for nearly all backbones was consistently observed with decision tree models, with the lowest at 72.04% for the Swin + HOG setup. The weakest performance among backbones was with VGG-16 + HOG + DT, which dropped to 71.09% and had the lowest AUC (69.50%). VGG 16 had the lowest peak among the six backbones (89.39% with LR), making it the least competitive feature extractor in terms of accuracy. However, it remained viable with linear or margin classifiers.

Performance trends consistently demonstrated that neural or linear classifiers performed the best, followed by margin classifiers, then ensemble trees, and finally DTs.

Table 11 presents a comparison of model accuracies without and with HOG features in the second and first experiments, respectively. Transformer-based models, Swin and ViT, maintained peak accuracies of 94.68% and 94.51%, respectively, indicating that adding HOG features did not improve their performance. Conversely, CNN-based models showed significant improvements with HOG features. DenseNet-201 increased from 89.81% to 91.13%, DenseNet-121 rose from 89.56% to 92.12%, MobileNet improved from 90.80% to 92.02%, and VGG-16 went from 84.86% to 89.39%. This indicates that HOG features enhance CNN feature representation, especially for those with moderate performance. In contrast, transformer models, which inherently capture spatial and structural details, do not benefit from HOG.

In the third experiment, we used the two transformers (Swin and ViT) alongside the four DL models for feature extraction. The outcomes of the 5-fold cross-validation for the five ML models, along with their respective evaluation metrics, are shown in Table 12 and Table 13.

**Figure 18:**
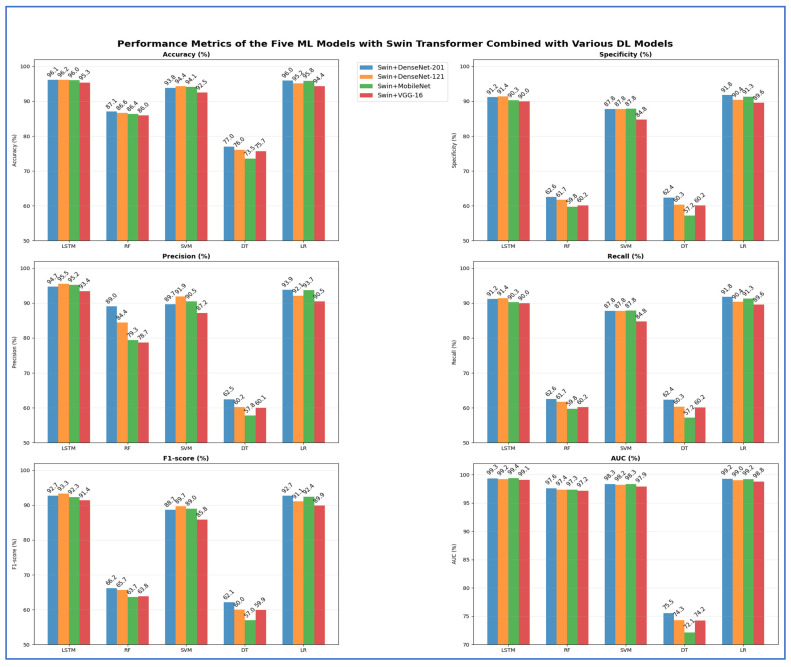
The performance metrics of the five ML approaches with Swin Transformer and DL models combinations.

Table 12 and Figure 18 reveal that models based on integrated deep representations, which combined the Swin transformer with various CNN backbones, achieved excellent classification results. Notably, when temporal and margin-based learners were employed, the Swin + DenseNet-201 with LSTM model attained the highest overall accuracy of 96.10% and an AUC of 99.33%. This was closely followed by Swin + DenseNet-121 with LSTM, achieving 96.17% accuracy and a 99.17% AUC. This confirms that the DenseNet backbones effectively retain detailed features of teeth and lesions after fusion. The compact model Swin + MobileNet with LSTM also demonstrated strong performance, achieving 96.05% accuracy and 99.37% AUC, illustrating that even lightweight CNNs can capture important structural and pathological information. Among non-sequential learners, Swin + DenseNet-121 paired with SVM and LR achieved competitive accuracy (94.36% and 95.15%, respectively, with AUC > 99%), suggesting that the fused embeddings were well-structured for decision boundaries and probabilistic separation. The analysis showed that the LSTM-based fusion pipelines—Swin combined with DenseNet-201, DenseNet-121, and MobileNet—achieved the highest accuracies. At the same time, tree-based models like DT showed the lowest generalization, particularly in specificity and AUC. Overall, the study confirmed that the DenseNet-201 with LSTM and DenseNet-121 with LSTM fusion models were the most precise, with all LSTM variants exceeding 95% accuracy, establishing them as the top-performing approaches in the experiment.

**Table 12:** The mean cross-validation scores for the five ML models with Swin and DL models combinations.

Deep Features	ML	Accuracy (%)	Specificity (%)	Precision (%)	Recall (%)	F1-Score (%)	AUC (%)
Swin + DenseNet-201	LSTM	96.10	91.24	94.68	91.24	92.72	99.33
	RF	87.10	62.56	89.03	62.56	66.22	97.58
	SVM	93.84	87.80	89.68	87.80	88.65	98.33
	DT	77.01	62.35	62.49	62.35	62.15	75.53
	LR	95.95	91.77	93.86	91.77	92.74	99.23
Swin + DenseNet-121	LSTM	96.17	91.44	95.53	91.44	93.29	99.17
	RF	86.63	61.73	84.40	61.73	65.67	97.36
	SVM	94.36	87.81	91.94	87.81	89.68	98.21
	DT	76.04	60.34	60.25	60.34	60.00	74.29
	LR	95.15	90.40	92.09	90.40	91.09	99.03
Swin + MobileNet	LSTM	96.05	90.33	95.23	90.33	92.35	99.37
	RF	86.40	59.77	79.35	59.77	63.66	97.32
	SVM	94.08	87.84	90.45	87.84	89.02	98.35
	DT	73.55	57.20	57.77	57.20	56.98	72.13
	LR	95.7992	91.28841	93.73838	91.28841	92.41986	99.17529
Swin + VGG-16	LSTM	95.30	89.99	93.44	89.99	91.43	99.08
	RF	85.96	60.18	78.69	60.18	63.81	97.15
	SVM	92.52	84.77	87.15	84.77	85.83	97.92
	DT	75.67	60.16	60.06	60.16	59.91	74.22
	LR	94.38	89.60	90.51	89.60	89.91	98.75

**Figure 19:**
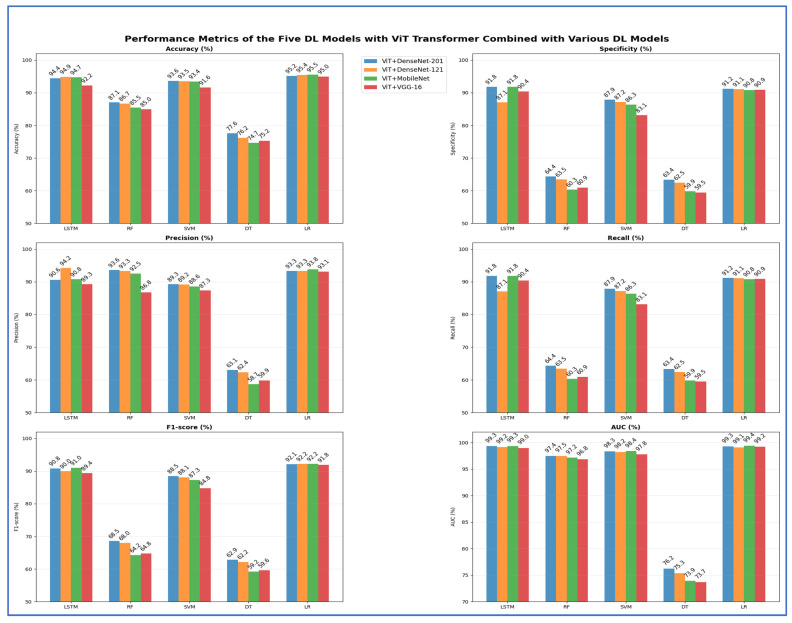
The performance metrics of the five ML approaches with ViT Transformer and DL models combinations.

From Table 13 and Figure 19, the highest peak accuracy was 95.50%, achieved by the ViT + MobileNet with LR combination. The lowest accuracy noted was 74.70%, produced by ViT + MobileNet with DT. The fusion using ViT + DenseNet-121 with LR also demonstrated strong accuracy at 95.40%, making it one of the leading models, closely followed by ViT + DenseNet-201 with LR, which achieved 95.18%. Both DenseNet fusion backbones demonstrated high stability with linear and neural classifiers, particularly LSTM and LR, achieving accuracies consistently above 94%. SVM remained competitive in the transformer–CNN fusion domain, achieving accuracies of 91.60–93.64%, while ensemble tree models like RF reached moderate peaks (85–87%) but showed apparent limitations in recall (60–64%). DT models performed worst across nearly all backbones, with accuracies ranging from 74.70% to 77.58% and significantly lower AUC values (~73–76%) compared to neural and linear classifiers. The highest overall AUC was 99.40% for MobileNet with LR fusion, indicating that linear classifiers effectively leveraged the fused deep features. In contrast, shallow trees struggled to maintain the discriminative structure of the feature space.

**Table 13:** The mean cross-validation scores for the five ML models with ViT and DL models combinations.

Deep Features	ML	Accuracy (%)	Specificity (%)	Precision (%)	Recall (%)	F1-Score (%)	AUC (%)
ViT + DenseNet-201	LSTM	94.41	91.79	90.59	91.79	90.78	99.30
	RF	87.10	64.37	93.64	64.37	68.55	97.43
	SVM	93.64	87.86	89.30	87.86	88.46	98.33
	DT	77.58	63.38	63.09	63.38	62.85	76.20
	LR	95.18	91.19	93.30	91.19	92.11	99.27
ViT + DenseNet-121	LSTM	94.88	87.06	94.25	87.06	89.98	99.15
	RF	86.70	63.48	93.34	63.48	68.00	97.49
	SVM	93.54	87.20	89.18	87.20	88.07	98.22
	DT	76.16	62.46	62.37	62.46	62.18	75.35
	LR	95.40	91.11	93.32	91.11	92.16	99.10
ViT + MobileNet	LSTM	94.71	91.76	90.77	91.76	91.03	99.35
	RF	85.46	60.32	92.49	60.32	64.23	97.16
	SVM	93.39	86.33	88.59	86.33	87.26	98.37
	DT	74.70	59.85	58.69	59.85	59.16	73.94
	LR	95.50	90.81	93.78	90.81	92.17	99.40
ViT + VGG-16	LSTM	92.17	90.43	89.27	90.43	89.40	98.98
	RF	84.99	60.89	86.76	60.89	64.79	96.84
	SVM	91.60	83.10	87.33	83.10	84.78	97.75
	DT	75.24	59.47	59.87	59.47	59.57	73.68
	LR	94.95	90.89	93.09	90.89	91.85	99.19

The third experiment demonstrated that both feature-extraction methods—Swin-based and ViT-based—performed well, with noticeable differences in the models’ ability to generalize across classifiers. As shown in the third experiment, among the Swin fusion models, the Swin + DenseNet-121 with LSTM model achieved the highest accuracy (96.17%), whereas the Swin + DenseNet-201 with DT setup showed the lowest accuracy (77.01%). In the ViT fusion models, the ViT + MobileNet with LR model attained the highest accuracy (95.50%), while the ViT + DenseNet-121 with DT model had one of the lowest accuracies (76.16%), and the ViT + VGG-16 with RF also performed poorly at 84.99%.

In the fourth experiment, we combined the DL models and the two transformers for deep feature extraction with HOG for handcrafted feature detection, again employing ML techniques as classifiers. The results of the 5-fold cross-validation for the five ML models, along with their respective evaluation metrics, are presented in Table 14 and Table 15.

**Figure 20:**
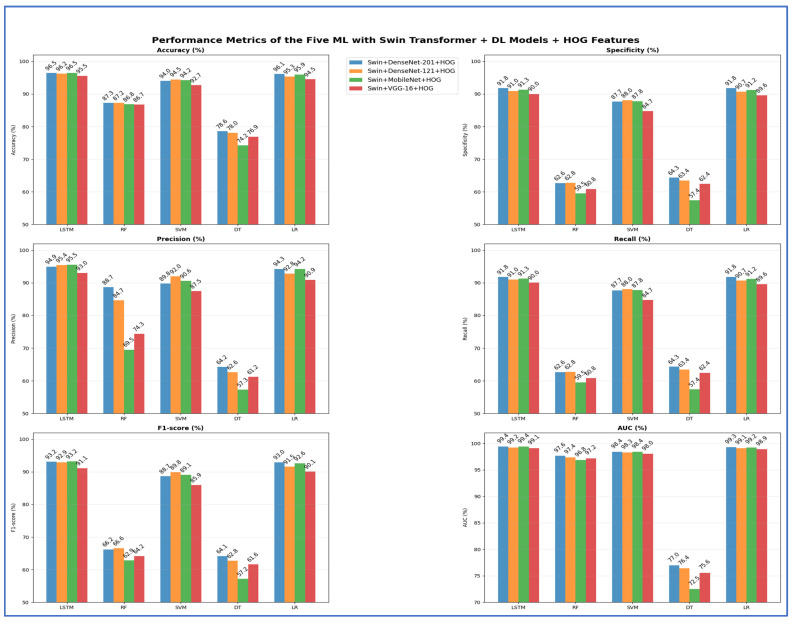
The performance metrics of the five ML approaches with DL models, Swin Transformer, and HOG combinations.

From Table 14 and Figure 20, the highest peak training accuracy had been 96.47%, achieved by the Swin + DenseNet-201 + HOG with LSTM configuration, followed very closely by Swin + MobileNet + HOG with LSTM at 96.45% and Swin + DenseNet-121 + HOG with LSTM at 96.20%. Linear classifiers with the fusion, particularly Swin + DenseNet-121 + HOG with LR (95.33%) and Swin + DenseNet-201 + HOG with LR (96.10%), had also delivered robust and stable performance, with AUC values exceeding 99.27%. Margin-based models such as the SVM had remained consistently competitive across all backbones (peaking between 92.67% and 94.48%) but had stayed slightly below the LSTM and LR peaks in accuracy. Ensemble trees, such as RF, demonstrated moderate performance across all backbones (86.70–87.30% accuracy), yet struggled with recall and specificity (ranging from 59 to 62%, depending on the fusion). DT had the lowest overall accuracy (74.22% with Swin + MobileNet + HOG) and showed the weakest discriminative performance, with AUC values declining sharply (72.47–76.99%) relative to neural and linear classifiers.

Overall, Swin transformer + CNN + HOG feature fusion backbones showed exceptional robustness when classified using neural sequence fusion (LSTM) or a linear decision boundary (LR). In contrast, shallow tree models consistently produced the most significant degradation in accuracy and separability.

**Table 14:** The mean cross-validation scores for the five ML models with DL models, Swin, and HOG combinations.

Deep Features	ML	Accuracy (%)	Specificity (%)	Precision (%)	Recall (%)	F1-Score (%)	AUC (%)
Swin + DenseNet-201 + HOG	LSTM	96.47	91.76	94.92	91.76	93.15	99.38
	RF	87.30	62.64	88.68	62.64	66.22	97.64
	SVM	94.03	87.71	89.82	87.71	88.65	98.38
	DT	78.60	64.32	64.22	64.32	64.12	76.99
	LR	96.10	91.85	94.27	91.85	92.95	99.27
Swin + DenseNet-121 + HOG	LSTM	96.20	90.95	95.43	90.95	92.91	99.21
	RF	87.22	62.78	84.67	62.78	66.58	97.36
	SVM	94.48	88.03	91.99	88.03	89.83	98.28
	DT	78.05	63.44	62.60	63.44	62.76	76.40
	LR	95.33	90.65	92.78	90.65	91.55	99.10
Swin + MobileNet + HOG	LSTM	96.45	91.30	95.49	91.30	93.17	99.39
	RF	86.85	59.51	69.55	59.51	62.87	96.81
	SVM	94.21	87.81	90.63	87.81	89.10	98.39
	DT	74.22	57.42	57.32	57.42	57.16	72.47
	LR	95.90	91.18	94.25	91.18	92.61	99.22
Swin + VGG-16 + HOG	LSTM	95.50	90.04	92.97	90.04	91.14	99.10
	RF	86.73	60.80	74.30	60.80	64.18	97.17
	SVM	92.67	84.71	87.46	84.71	85.91	98.00
	DT	76.91	62.40	61.19	62.40	61.59	75.57
	LR	94.53	89.56	90.89	89.56	90.07	98.88

**Figure 21:**
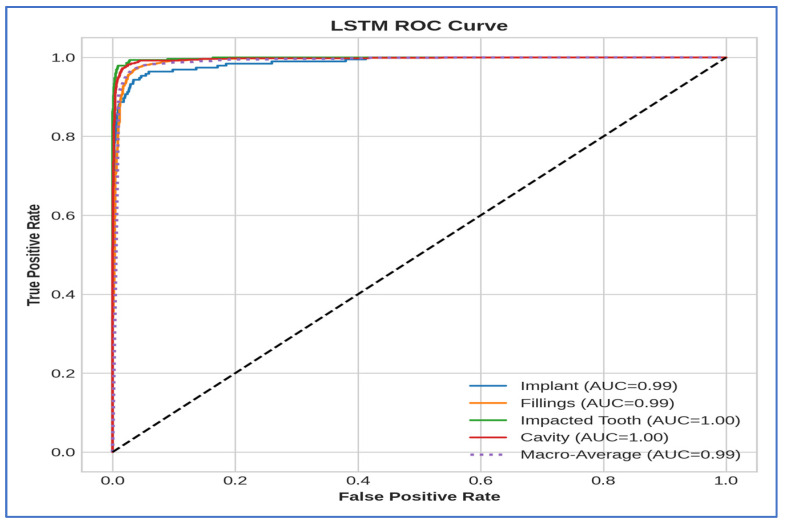
The ROC curve of the LSTM classifier with DenseNet-201 DL models, Swin Transformer, and HOG combinations.

Figure 21 displays the Receiver Operating Characteristic (ROC) curve for the LSTM. The findings indicated that the model maintained consistently high performance across all categories. Notably, the Implant and Fillings classes achieved an AUC of 0.99, signifying excellent classification capability. The Impacted Tooth and Cavity classes showed perfect or nearly perfect performance, with each achieving an AUC of 1.00, indicating exceptional ability to differentiate these conditions from other categories. Additionally, the macro-average ROC curve yielded an AUC of 0.99, highlighting the LSTM model’s robustness and reliability across all dental categories. The ROC curves were located near the top-left corner of the graph, with high true-positive rates at very low false-positive rates, confirming the effectiveness of the proposed feature fusion and classification framework in accurately identifying dental pathologies.

**Figure 22:**
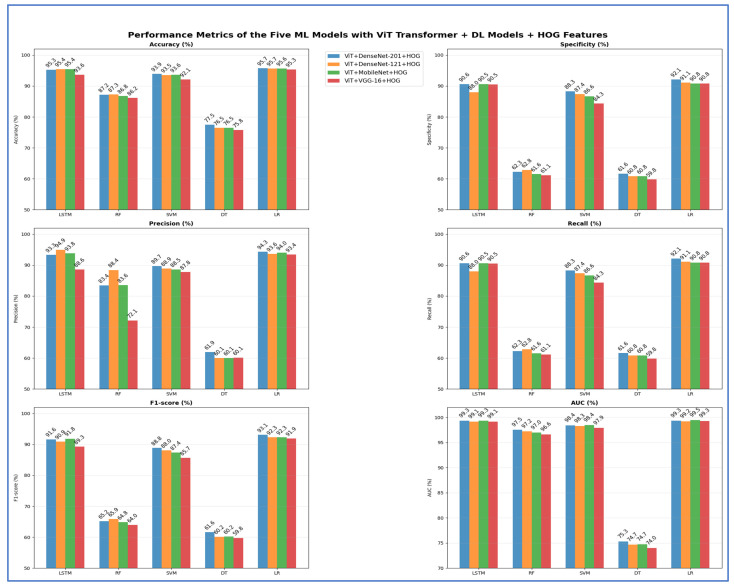
The performance metrics of the five ML approaches with DL models, ViT Transformer, and HOG combinations.

Table 15 and Figure 22 demonstrate that the ViT + DenseNet-201 + HOG achieved the highest overall accuracy of 95.72% with the LR model. Similarly, high performance was observed in the ViT + DenseNet-121 + HOG with LR model (95.65%), and in both the ViT + MobileNet + HOG with LSTM and the ViT + DenseNet-121 + HOG with LSTM models (95.40%). This indicates that linear and sequence models effectively utilized the fused feature structure.

The lowest accuracies were consistently found with DT classifiers, with the global low of 75.77% for ViT + VGG-16 + HOG with DT, followed closely by ViT + DenseNet-121 + HOG with DT (76.49%) and ViT + MobileNet + HOG with DT (76.49%). This showed that shallow decision trees were less effective at preserving the discriminative gradients of the fused embeddings than neural or margin-based models.

SVM was the most competitive non-neural classifier, achieving 93.89% (ViT + DenseNet-201 + HOG) and 92.10% (ViT + VGG-16 + HOG), showing a strong balance between precision and recall. RF ensembles achieved moderate accuracy peaks (~86–87%), but their recall and specificity were significantly lower (~61–63%), indicating difficulty in reliably detecting positive and negative cases despite high AUC values (~96–97%).

AUC trends revealed that transformer-driven fusion backbones with LR or LSTM achieved near-perfect separability (~99.29–99.45%), while DT classifiers had the weakest AUC values (~73.99–75.27%), reinforcing their limited discrimination.

Overlap in the peak ranges of LSTM and LR indicated that models combining feature-rich transformer-CNN fusion were more effective for temporal or continuous decision modeling. In contrast, DT classifiers performed poorly across almost all metrics and backbones.

**Table 15:** The mean cross-validation scores for the five ML models with DL models, ViT, and HOG combinations.

Deep Features	ML	Accuracy (%)	Specificity (%)	Precision (%)	Recall (%)	F1-Score (%)	AUC (%)
ViT + DenseNet-201 + HOG	LSTM	95.28	90.60	93.35	90.60	91.64	99.30
	RF	87.17	62.26	83.41	62.26	65.19	97.52
	SVM	93.89	88.29	89.65	88.29	88.85	98.38
	DT	77.45	61.64	61.92	61.64	61.59	75.27
	LR	95.72	92.10	94.33	92.10	93.09	99.34
ViT + DenseNet-121 + HOG	LSTM	95.40	87.99	94.89	87.99	90.89	99.14
	RF	87.27	62.81	88.42	62.81	65.88	97.23
	SVM	93.54	87.36	88.88	87.36	88.02	98.28
	DT	76.49	60.80	60.06	60.80	60.16	74.66
	LR	95.65	91.09	93.61	91.09	92.26	99.17
ViT + MobileNet + HOG	LSTM	95.40	90.55	93.82	90.55	91.84	99.35
	RF	86.78	61.58	83.56	61.58	64.81	96.97
	SVM	93.59	86.63	88.55	86.63	87.40	98.43
	DT	76.49	60.79	60.06	60.79	60.24	74.74
	LR	95.63	90.77	94.04	90.77	92.26	99.45
ViT + VGG-16 + HOG	LSTM	93.64	90.52	88.58	90.52	89.31	99.13
	RF	86.18	61.14	72.14	61.14	63.95	96.57
	SVM	92.10	84.30	87.80	84.30	85.67	97.88
	DT	75.77	59.78	60.11	59.78	59.76	73.99
	LR	95.30	90.76	93.38	90.76	91.91	99.29

Table 16 presents a comparison of model accuracies without and with HOG features in the third and fourth experiments, respectively. The combination of Swin with DenseNet-201 and LSTM increased from 96.10% to 96.47%. Similarly, Swin with DenseNet-121 and LSTM saw a slight improvement from 96.17% to 96.20%. Swin, paired with MobileNet and LSTM, increased from 96.05% to 96.45%. Among the ViT-based models, DenseNet-121 with LR improved from 95.40% to 95.65%, DenseNet-201 with LR rose from 95.18% to 95.72%, and MobileNet with LR increased from 95.50% to 95.63%. These findings suggest that HOG contributed positively by providing additional structural and edge details, enhancing feature representation, and slightly boosting classification accuracy for both CNN and transformer-based architectures.

Based on the four experiments, we found that the model using Swin + DenseNet-201 + HOG with LSTM achieved the highest accuracy of 96.47%. This indicates that neural and linear classifiers effectively leveraged the combined features of the transformer, CNN, and HOG, especially when Swin + DenseNet-201 + HOG was paired with LSTM. In contrast, tree classifiers, particularly decision trees (DTs), performed less effectively and showed the most significant drop in performance.

### 4.1. Component-Wise Performance Analysis

In an ablation study, parts of the model or groups of features were systematically removed to assess their impact on diagnostic ability and overall performance. In DL experiments using dental imaging, ablation analysis identified which convolutional blocks, attention layers, or handcrafted features were most effective at detecting subtle structural anomalies, such as enamel defects, periodontal bone loss, and dental lesion boundaries [32]. Our research involved four experiments that used an ablation engine to evaluate the contribution of each feature set—transformers only, CNNs only, dual-fusion, and full triple-fusion—under consistent experimental conditions. Table 17 presents the results of the ablation.

Table 17 presents that using only CNN features with an LSTM classifier resulted in an accuracy of 90.80%. When Swin features were used exclusively with LSTM, accuracy improved to 94.68%. The combination of CNN and ViT features with a logistic regression (LR) classifier achieved 94.51%. Combining CNN with HOG features and using LSTM slightly increased the accuracy to 92.12%. Pairing HOG and Swin features with LSTM also resulted in 94.68% accuracy, while HOG and ViT features with LR achieved 94.51%.

Additional combinations showed better results: CNN paired with Swin features and LSTM achieved 96.17% accuracy, and CNN paired with ViT features and LR achieved 95.50% accuracy. The combination of CNN, HOG, and Swin features with LSTM achieved the highest accuracy of 96.47%, while CNN, HOG, and ViT features with LR resulted in 95.72%.

Overall, the findings suggested that using multiple feature fusions, especially CNN and Swin with LSTM, consistently enhanced classification performance. Including ViT features with LR also showed strong accuracy but was slightly less effective than the CNN–Swin–LSTM combination.

In Addition, we presented the effects of different preprocessing steps, such as CLAHE. Table 18 shows the results of DenseNet-201 + Swin + HOG + LSTM framework without CLAHE. The cross-validation results for the Swin + DenseNet-201 + HOG + LSTM model, without CLAHE, showed consistently strong and stable performance across all 5 folds. The model achieved an impressive average accuracy of 97.19%, with fold-wise accuracies ranging from 96.83% to 97.73%, indicating solid generalization and minimal performance variability. Both specificity and recall averaged 92.93%, confirming the model’s reliable ability to correctly identify negative cases while maintaining strong sensitivity to actual disease instances. The average precision reached 96.02%, reflecting a low false-positive rate, while the mean F1-score of 94.26% highlighted a well-balanced trade-off between precision and recall. Notably, the AUC values remained exceptionally high across all folds (99.04–99.54%), yielding an average AUC of 99.25%, which indicated excellent class separability and strong discriminative capability of the hybrid feature representation.

Overall, these results confirmed that the proposed hybrid framework delivered stable, high diagnostic performance even without contrast enhancement, underscoring the effectiveness of combining deep and handcrafted features with LSTM-based classification for X-ray image analysis.

The lower performance observed with CLAHE (96.47%) compared to the non-CLAHE setting can be explained by the interaction between contrast enhancement and deep feature learning. While CLAHE is commonly used to boost visual contrast in medical imaging, our findings indicate that its straightforward integration into DL pipelines might be detrimental. Pre-trained CNNs inherently encode natural image intensity statistics from large-scale datasets. CLAHE modifies these statistics by applying local histogram equalization, thus disrupting feature transfer and harming classification performance.

Additionally, dental radiographs often feature diagnostically important low-contrast patterns. Over-enhancing contrast can obscure subtle grayscale transitions and amplify noise and artifacts, causing the network to focus on irrelevant details. This accounts for the decreased accuracy and calibration observed when CLAHE was applied universally.

However, when used selectively as a stochastic augmentation during training, CLAHE enhanced model robustness and generalization. This implies that contrast enhancement is beneficial not as a fixed preprocessing step but as a method to promote invariance to local intensity variations.

These insights are consistent with recent studies showing that visually enhanced images do not necessarily improve deep learning performance. Therefore, contrast enhancement should be carefully managed and empirically tested rather than assumed to be advantageous.

### 4.2. Experimental Results: Statistical Validation

The paired *t*-test is a statistical method used to compare the means of two related or matched samples to see if there are significant differences between them. This test is useful when measurements are taken from the same individuals or units under two conditions, such as in before-and-after studies, matched pairs, or repeated measures. The core idea of the paired *t*-test is to account for variability between subjects by focusing on the differences within each pair, thus increasing statistical power compared to independent *t*-tests. The test calculates the average difference between paired observations and tests whether it is significantly different from zero, accounting for the variability of these differences and the sample size. It assumes that the differences between pairs follow a normal distribution and that the pairs are randomly selected from the population. Table 19 shows the results of the paired *t*-test for accuracy.

Table 19 presents a paired *t*-test to assess the significance of differences in accuracy between LSTM and other classifiers (LR and SVM) across various deep feature backbones. For the Swin + DenseNet-201 + HOG backbone, LSTM surpassed LR, with a mean accuracy of 96.47% compared to 96.10%, a t-statistic of 8.33, and a *p*-value of 0.0012, indicating a significant difference. Likewise, LSTM significantly outperformed SVM (mean 94.43%), with a *t*-statistic of 24.68 and a *p*-value of 0.0001.

For the Swin + DenseNet-121 + HOG backbone, LSTM achieved a mean accuracy of 96.20%, significantly higher than LR (95.33%) and SVM (94.48%), with t-statistics of 21.45 and 19.77, and *p*-values of 0.0001, confirming statistical significance in both comparisons. In the Swin + MobileNet + HOG setup, LSTM again outperformed LR (96.45% vs. 95.90%) and SVM (96.45% vs. 94.21%), with t-statistics of 16.84 and 23.56, and *p*-values of 0.0002 and 0.0001, respectively, showing significant differences. Lastly, with the Swin + VGG-16 + HOG backbone, LSTM achieved a mean accuracy of 95.50%, significantly higher than LR (94.53%) and SVM (92.67%), with *t*-statistics of 18.17 and 28.32 and *p*-values of 0.0001 in both cases. Overall, the analysis confirmed that LSTM consistently outperformed both LR and SVM across all feature backbones, with statistically significant differences in accuracy for all comparisons.

A confidence interval (CI) is a range derived from sample data that is expected to encompass the true value of an unknown population parameter, such as the actual model accuracy across all potential future data, with a given level of confidence [33]. If we repeatedly draw samples from the same population and create a 95% CI for a parameter each time, 95% of these intervals would include the true parameter value, while the other 5% would not. Table 20 presents the CIs for the LSTM DL model’s performance metrics. From Table 20*,* the LSTM model exhibited strong performance across evaluation metrics, with high mean values and narrow CIs. The AUC was particularly noteworthy, demonstrating excellent discrimination. The accuracy was 96.47% with minimal variability (±0.66%), indicating consistent performance and a true accuracy range of 95.81% to 97.13%. AUC was outstanding at 99.38% (±0.31%), demonstrating stable performance across folds and a tight interval of 99.07% to 99.70%. F1-score was 93.15% (±0.99%), reflecting good consistency. Precision was 94.92% (±1.48%), with a CI of 93.44% to 96.40%, indicating a strong positive predictive value. Recall and Specificity were 91.76% (±1.81%), showing the most significant variability and suggesting a dependence on fold composition, yet remaining robust above 89.95%.

Consistently high lower bounds for CIs confirm model robustness, reducing the likelihood of chance high scores. The disparity between the tight AUC interval and wider recall/specificity intervals suggests stable overall ranking performance, though per-class performance varied more. All metrics maintained practical performance levels at their 95% lower confidence bounds. In summary, the LSTM model demonstrated statistically reliable performance and strong generalization. CIs ensure that reported metrics reflect the model’s actual performance rather than the data.

### 4.3. External Validation

External validation assesses a trained DL model’s performance on independent, unseen data from sources distinct from the training and test sets. This approach rigorously evaluates the model’s ability to generalize to real-world deployment conditions. For DL models, this involves evaluating the completed model architecture and weights on data that accurately reflects distribution changes, rather than merely using random splits of the same foundational dataset. External validation for DL models ensures robustness against distribution shifts, fosters clinical trust, and enables safe deployment. It addresses under-specification and adheres to regulatory and scientific rigor [34].

In our research, we conducted the external validation of the proposed hybrid framework using 3365 oral endoscopic images. These images were randomly split into three groups: a training dataset of 2019 images, a validation dataset of 673 images, and a test dataset of 673 images. The images in the training and validation sets were manually labeled. We integrated the DenseNet-201 DL model with the Swin Transformer to extract deep features, alongside HOG for handcrafted feature detection. For classification, we employed ML-LSTM, LR, RF, and DT techniques.

**Figure 23:**
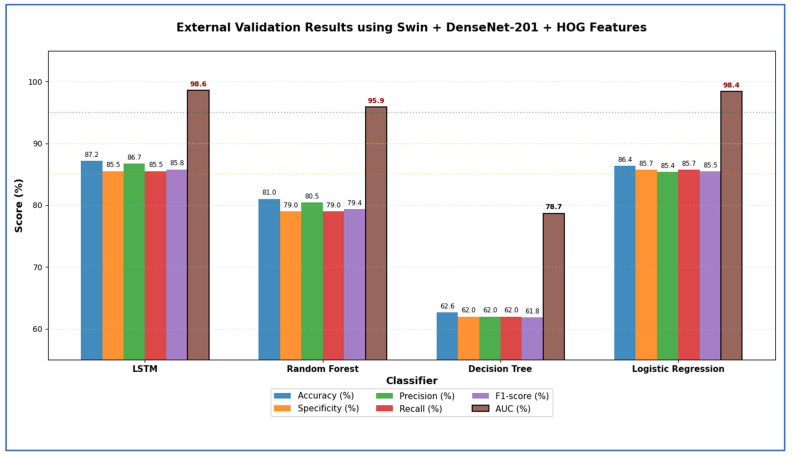
The results of the external validation.

Table 21 and Figure 23 present the results of the 5-fold cross-validation for these four ML models, along with their evaluation metrics. Table 21 shows that LSTM emerged as the top-performing model among all those evaluated. It demonstrated excellent classification performance, achieving 87.16% accuracy and balanced precision of 86.72% and recall of 85.51%, resulting in an F1-score of 85.78%. The model’s strong discrimination ability was highlighted by its outstanding AUC of 98.61%. With a specificity of 85.51%, it reliably identified true negatives, making it the most effective classifier for this feature combination.

RF exhibited moderate performance, which, although respectable, was considerably lower than that of the leading models. It achieved an accuracy of 81.03%, precision of 80.47%, recall of 78.97%, and an F1-score of 79.37%. The specificity of 78.97% indicated adequate but imperfect detection of true negatives. The AUC of 95.86% showed strong discriminative power, but the difference between the AUC and other metrics suggested potential calibration issues or class imbalance.

DT performed the worst among the models, indicating limited suitability for this feature set and classification task. It achieved the lowest accuracy at 62.64%, with uniformly low metrics across precision, recall, and specificity (all around 61.98%), resulting in an F1-score of 61.82%. Its AUC of 78.69% confirmed its inferior discriminative ability, implying the model struggled to capture meaningful patterns and likely suffered from significant overfitting.

LR stood out as the second-best performer, achieving competitive results despite its simplicity. It reached an accuracy of 86.38%, just below LSTM, with a precision of 85.39% and recall of 85.73%, yielding an F1-score of 85.46%. The model demonstrated excellent specificity (85.73%) and an impressive AUC of 98.39%, nearly matching LSTM’s discriminative capacity. This performance suggests that the deep features were highly linearly separable, allowing logistic regression to achieve results close to those of more complex architectures.

### 4.4. Computational Cost

The computational cost of a DL model refers to the resources required to train and deploy it, often quantified by training and inference durations, memory usage, energy consumption, and model size (number of parameters). This cost is crucial because it affects the feasibility, scalability, and practical use of DL systems, particularly in resource-limited environments such as clinical or industrial settings [35].

High computational demands can hinder model adoption due to increased hardware requirements, longer training times, higher energy consumption, and inference latency, which may be unacceptable for time-sensitive applications such as medical diagnostics or autonomous systems. Therefore, assessing computational cost alongside predictive performance enables a more balanced evaluation of DL models, facilitating informed decisions among accuracy, efficiency, sustainability, and deployment feasibility rather than focusing solely on metric superiority [35].

Table 22 shows the computational costs for the different combinations of DL, Swin, and HOG. The analysis of computational costs revealed that the Swin + DenseNet-201 + HOG model combined with the LSTM classifier was the most efficient setup. This model achieved an impressive 97.15% accuracy while maintaining the shortest total training time of 23.50 s among all high-performing models, along with a very low mean inference time per sample of 2.61 × 10^−5^ s. These results demonstrated an optimal balance between predictive accuracy and computational efficiency.

Although the Swin + MobileNet + HOG + LSTM model yielded slightly higher accuracy (97.35%), its training time and inference cost were somewhat higher, and the lightweight backbone introduced greater variability across classifiers. In contrast, DenseNet-201 provided more stable and discriminative deep feature representations, leading to consistently strong performance with lower computational demands when paired with LSTM.

Across all feature extractors, LSTM classifiers consistently outperformed LR, RF, DT, and SVM in accuracy while maintaining low inference latency, confirming their suitability for sequential modeling of hybrid deep features. SVM classifiers exhibited the highest computational cost, with significantly longer training and inference times, particularly for DenseNet-201 and VGG-16 backbones, making them impractical for real-time clinical use.

In conclusion, the findings confirmed that the Swin + DenseNet-201 + HOG + LSTM framework was the best-performing and most computationally efficient solution, making it the most suitable candidate for automated disease diagnosis from X-ray images in clinical settings.

Table 23 provides the cost analysis of feature extraction for the DenseNet-201 + Swin + HOG framework. Table 23 shows that DL components were the main contributors to computational load. Specifically, DenseNet-201 (139.40 s) and the Swin Transformer (108.16 s) accounted for the largest share of processing time, highlighting the significant computational complexity of extracting features from X-ray images using deep hierarchical and attention-based methods. In contrast, handcrafted HOG features required minimal computation (4.11 s), and their dimensionality reduction added only a slight overhead (0.67 s). Feature fusion was computationally insignificant (0.01 s). The total feature extraction time was 4667.35 s, indicating that repeated forward passes and batch processing in deep networks contributed significantly to the overall cost. Despite this extended extraction time, the results showed that the primary driver of the additional computational expense was deep feature learning, which was justified by the enhanced diagnostic accuracy achieved by the hybrid framework.

### 4.5. Discussion of Results in Light of Recent Advances

**Figure 24:**
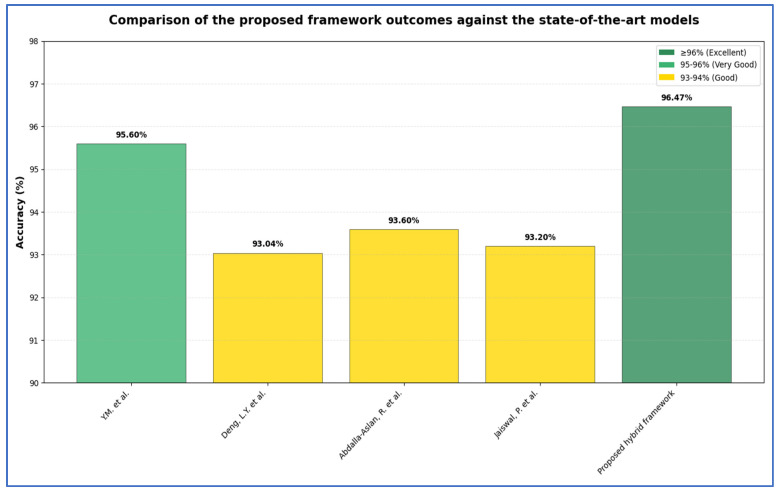
The comparison of the proposed framework’s outcomes with state-of-the-art models [2,16,17,19].

The effectiveness of the proposed hybrid framework for detecting dental disorders was assessed against several recent studies using X-ray imaging, as shown in Table 24 and Figure 24. This approach, which integrates Swin transformer, DenseNet-201, and HOG features, achieved a peak accuracy of 96.47%, outperforming all previous methods considered in this comparison.

Earlier research primarily utilized traditional DL models. For instance, Y.M. et al. [2] employed a combination of the Swin Transformer, MobileNet-V2, and a bagging ensemble, achieving an accuracy of 95.6%, which was slightly lower than that of the proposed framework. Similarly, Jaiswal et al. [19] used ResNet50-V2, ResNet101-V2, and MobileNet variants, achieving 93.2% accuracy, while Deng et al. [16] reported 93.04% accuracy with a standard CNN. These findings suggest that while conventional CNN-based models and lightweight networks like MobileNet perform well, they are surpassed by hybrid architectures that integrate advanced transformer architectures with deep convolutional networks.

Regarding precision, the proposed framework also achieved impressive results, consistently exceeding 94.92% in tests, comparable to Baydar et al. [14], who reported a precision of 94.91% with a U-Net architecture. Other studies, such as Yüksel et al. [5] and Ghaznavi Bidgoli et al. [18], reported lower precision values (89.4% and 92%, respectively), underscoring the hybrid approach’s enhanced discriminative capacity. The proposed hybrid framework not only achieved high accuracy and precision but also demonstrated strong discriminative ability, as evidenced by its superior AUC of 99.38% compared to previous methods. Lee et al. [15] documented an AUC of 0.917 (95% CI 0.860–0.975) for a CNN-based approach. In contrast, the hybrid model’s integration of the Swin Transformer, DenseNet-201, and HOG features enabled it to better differentiate between positive and negative cases, thereby enhancing its ability to distinguish between healthy and diseased dental structures.

The combination of transformer-based features (Swin) with DenseNet-201 and handcrafted HOG descriptors likely enhanced feature representation, capturing both overarching structural patterns and detailed textural nuances in dental X-rays. This multimodal fusion enabled superior performance compared to individual network models such as AlexNet, VGG-16, and conventional CNNs (You et al. [6]; Krois et al. [20]).

Furthermore, the framework’s blend of DL and handcrafted features resulted in greater resilience across varying X-ray imaging conditions. Prior methods, especially those that depended solely on CNNs or lighter networks like MobileNet, AlexNet, or VGG-16, were more sensitive to changes in image quality and subtle structural differences, which could affect generalization. The hybrid model’s ability to capture global contextual information (through the Swin Transformer and DenseNet-201) and detailed texture features (through HOG) improved its performance across a wide range of dental X-rays.

These results indicate that the proposed model not only surpasses existing models in raw accuracy but also provides more reliable detection, which is essential for early diagnosis and clinical decision-making. Combining advanced transformer architectures with traditional CNNs and handcrafted descriptors presents a promising avenue for future research in automated dental disorder detection. In summary, the proposed hybrid framework demonstrated that integrating transformer-based architectures with deep CNNs and handcrafted features can significantly improve the accuracy and precision of dental disorder detection in X-ray imaging, setting a new standard for future research in this field.

Optimizing our model’s performance has been a priority; however, achieving 100% accuracy remains difficult. This is due to several factors, including variability in imaging quality, differences in image scanners, and inherent limitations within the dataset. Additional challenges arise from noise, artifacts, and interobserver variability. Despite these obstacles, our model demonstrates competitive performance when compared to existing methods, and we have thoroughly assessed its accuracy using standard metrics. The proposed DL model has shown potential to outperform other recent classifiers, particularly following parameter tuning. We believe our approach lays a strong foundation for further advancements in this field.

### 4.6. Limitations and Future Work

While the study shows promising results, several limitations must be noted. First, the feature fusion method combined high-dimensional outputs from the transformer and deep CNN backbones. This increased computational demands and memory usage, making it less suitable for low-resource devices or real-time clinical applications. Second, using an LSTM for sequential classification treated these fused vectors as temporal data, even though the features were not inherently time dependent. Third, the framework was tested on only one radiography dataset, which, though adequate for an initial evaluation, was relatively small and may not fully reflect the broad variability across patients and institutions in large-scale clinical practice. Moreover, differences in imaging protocols, scanner brands, and reconstruction settings can significantly affect image appearance and feature distribution, potentially compromising the model’s robustness when applied to external datasets. Additionally, real-world clinical images often exhibit noise, motion artifacts, and other degradations that are not adequately represented in controlled or publicly available datasets. These issues may restrict the framework’s generalizability to new clinical environments.

Future efforts will focus on enhancing efficiency and clinical relevance. Planned improvements include using learnable or compressed fusion strategies, such as attention-based methods or dimensionality reduction, to minimize redundancy while preserving diagnostic information. The pipeline will also aim to incorporate classifiers that align with the non-temporal nature of the fused features, like transformers for feature interaction modeling.

## 5. Conclusions

In this paper, we introduce a novel hybrid DL framework that combines HOG, a handcrafted descriptor, with DenseNet-201 and the Swin Transformer for feature extraction. This integration enhances classification performance by fusing semantic, structural, and texture-based information. An LSTM network was used for classification by learning sequential relationships among the fused features. The hybrid model was explicitly designed to help clinicians in precisely and efficiently detecting dental diseases at an early stage. It aims to reduce diagnostic workload, minimize differences between observers, lower costs, and enhance patient results. An ablation engine was included to evaluate the contribution of each feature set—transformers only, CNNs only, dual-fusion, and full triple-fusion—under consistent experimental conditions. We conducted four extensive experiments using 5-fold cross-validation, demonstrating the robustness of the pipeline and highlighting the synergistic effect of combining deep and handcrafted features. The DRAD dataset was used, with preprocessing steps including resizing, normalization, CLAHE enhancement, and image cropping. The proposed LSTM-based hybrid model achieved 96.47% accuracy, 91.76% specificity, 94.92% precision, 91.76% recall, and 93.14% F1-score. The proposed modular framework offers flexibility, interpretability, and strong empirical performance, making it suitable for various image-based recognition applications and serving as a reproducible framework for future research on hybrid feature fusion and sequence-based classification.

## Figures and Tables

**Table 1:** The distribution of the training set of the DRAD dataset.

Class	Count
Fillings	2609
Implants	910
Impacted	301
Cavities	203
Total	4023

**Table 2:** A comparative analysis of the five ML models.

Criterion	LSTM	RF	SVM	DT	LR
Model Type	Sequential Neural Network	Ensemble Machine Learning	Kernel-Based Machine Learning	Classical Machine Learning	Classical Statistical Machine Learning
Core Principle	Utilizes memory cells and gated updates for learning long-term dependencies	Combines multiple decision trees using bagging for voting/averaging	Finds maximum-margin hyperplane; uses kernels for mapping non-linear data	Splits data using rules to maximize information gain or reduce impurity	Models class probability with a logistic linear function
Input Type	Time-series or sequences (e.g., pixel sequences, temporal signals)	Tabular or vectorized features	Tabular or vectorized features	Tabular or vectorized features	Tabular or vectorized features
Architecture	Recurrent chain of cells with hidden and cell states (utilizes forget, input, and output gates)	Multiple independent trees trained on random subsets of data and features	No defined neural architecture; operates in transformed feature space	Single tree of decision nodes and leaf predictions	Single weight vector plus bias
Feature Handling	Automatically learns sequential features (no need for manual engineering)	Handles mixed features; selects random features internally	Requires pre-computed features; sensitive to feature scales unless normalized	Selects features at nodes; not sensitive to scale	Requires normalized/clean linear features
Overfitting Control	Dropout, recurrent dropout, early stopping, gradient clipping, and gating regularization	Limits on tree depth, number of trees, bootstrap sampling, and randomness in the feature subspace	Regularization (C, margin), kernel choice, gamma; requires tuning	Pruning depth, minimum samples per leaf, post-pruning	L1/L2 regularization (Ridge/Lasso), threshold tuning
Interpretability	Low (black-box temporal model)	Medium (feature importance available but less transparent in the ensemble)	Medium–Low (boundary not easily interpretable)	High (explicit decision rules)	High (coefficients directly map feature to log-odds impact)
Compute Cost	High (back-propagation through time, sequential dependency)	Medium–High (many trees but parallelizable)	High on large data (kernel matrix scales poorly)	Low–Medium	Very Low
Scalability	Medium–Low (slow on very long sequences)	High (parallel training/inference)	Medium–Low on large datasets	High	Very High
Typical Applications	NLP, speech, video frames, ECG/EEG signals, sensor monitoring	Finance, medical tabular models, image, and engineered features, multi-class tasks	Image recognition with HOG/LBP, medical CAD systems, text with TF-IDF, bio-signals	Expert systems, clinical rule modeling, education, diagnostics	Risk prediction, binary medical classification, disease screening baselines
Role in Dental Disease Diagnosis	Models temporal signals (e.g., chewing acoustics, longitudinal patient metrics, sequential patches of X-ray scans)	Classifies radiomic/texture features for caries, bone loss, lesion prediction; provides importance per region.	Classifies engineered intraoral/X-ray features (e.g., HOG texture) for lesion/non-lesion separation.	Provides rule-based diagnosis or triage logic (severity paths, symptom thresholds)	Probability modeling using clinical metrics or extracted image features for disease/no-disease decisions
Advantages	Captures long-range sequential context, adaptive memory, and strong with temporal progression analysis	Reduces variance via ensembling, handles non-linear relations, is robust to noise, has no need for feature scaling, provides importance	Works well on small medical datasets, powerful non-linear boundaries, and effective with high-dimensional features	Fast inference, human-readable decisions, minimal preprocessing, easy deployment	Extremely efficient, provides uncertainty/probabilities, highly interpretable, strong baseline for medical/dental CAD
Disadvantages	Requires large data, slow training, risk of vanishing/exploding gradients, low transparency	Larger memory for many trees, slower than single models, harder rule tracing	Poor scaling on very large data, kernel heavy, sensitive to C/gamma tuning, needs feature engineering	Unstable to small data changes, tends to overfit if unpruned, limited non-linear complexity	Limited non-linear capacity, struggles if decision is not approximately linear, feature dependent

**Table 3:** The experiments’ hyperparameters.

Parameter	Value
Input image size	224 × 224
Color channels	3
Pooling operation	AAP
Output feature vector dimension	512
Preprocessing	Normalization + Resize + Cropping
Optimizer	Not applicable (model frozen; no training performed)
Loss function	Not applicable (feature extraction only; no backpropagation)

**Table 10:** The mean cross-validation scores for the five ML models using DL models or transformers with HOG.

Deep Features	ML	Accuracy (%)	Specificity (%)	Precision (%)	Recall (%)	F1-Score (%)	AUC (%)
Swin + HOG	LSTM	94.68	87.75	92.50	87.75	89.76	98.97
	RF	86.48	58.82	69.15	58.82	62.28	95.17
	SVM	92.59	84.76	87.16	84.76	85.84	97.94
	DT	72.04	53.39	52.60	53.39	52.76	70.06
	LR	94.01	88.35	90.21	88.35	89.18	98.66
ViT + HOG	LSTM	88.04	88.49	80.87	88.49	82.94	98.56
	RF	84.81	62.29	91.08	62.29	66.96	95.95
	SVM	91.13	81.80	86.77	81.80	83.79	97.57
	DT	73.33	61.16	59.14	61.16	59.91	74.18
	LR	94.51	90.78	92.22	90.78	91.36	98.99
DenseNet-201 + HOG	LSTM	90.68	84.28	83.41	84.28	83.16	98.06
	RF	85.24	58.09	66.28	58.09	60.92	93.06
	SVM	89.56	77.84	83.88	77.84	80.33	96.84
	DT	71.54	52.95	52.48	52.95	52.50	69.69
	LR	91.13	83.39	85.45	83.39	84.35	97.88
DenseNet-121 + HOG	LSTM	92.12	81.23	89.42	81.23	84.55	97.85
	RF	84.51	56.95	65.85	56.95	59.90	93.23
	SVM	88.44	75.17	82.44	75.17	78.08	96.29
	DT	71.29	53.79	52.63	53.79	52.91	70.22
	LR	90.48	82.68	84.75	82.68	83.55	97.42
MobileNet + HOG	LSTM	92.02	83.93	86.29	83.93	84.71	98.08
	RF	85.11	58.35	67.52	58.35	61.40	93.81
	SVM	90.13	79.05	84.33	79.05	81.25	97.08
	DT	71.27	53.77	52.77	53.77	53.10	70.04
	LR	91.08	83.11	84.06	83.11	83.41	97.63
VGG-16 + HOG	LSTM	87.10	72.01	79.29	72.01	73.90	95.86
	RF	83.94	56.61	64.67	56.61	59.33	92.36
	SVM	82.97	65.95	77.36	65.95	69.63	93.56
	DT	71.09	52.65	51.87	52.65	52.00	69.50
	LR	89.39	78.76	82.35	78.76	80.27	96.59

**Table 11:** A comparative performance between DL techniques and transformers with and without HOG.

Deep Model	Without HOG: Best ML Accuracy (%)	With HOG: Best ML Accuracy (%)	Δ (HOG − No HOG)
Swin	94.68	94.68	0.00
ViT	94.51	94.51	0.00
DenseNet-201	89.81	91.13	+1.32
DenseNet-121	89.56	92.12	+2.56
MobileNet	90.80	92.02	+1.22
VGG-16	84.86	89.39	+4.53

**Table 16:** A comparative performance between the combinations of DL techniques and Transformers with and without HOG.

Deep Feature Backbone	Classifier	Without HOG (%)	With HOG (%)	Δ (HOG − No HOG)
Swin + DenseNet-201	LSTM	96.10	96.47	+0.37
Swin + DenseNet-121	LSTM	96.17	96.20	+0.03
Swin + MobileNet	LSTM	96.05	96.45	+0.4
ViT + DenseNet-121	LR	95.40	95.65	+0.25
ViT + DenseNet-201	LR	95.18	95.72	+0.54
ViT + MobileNet	LR	95.50	95.63	+0.13

**Table 17:** The results of the ablation study.

CNN	HOG	Swin	ViT	Classifier	Accuracy (%)
True	False	False	False	LSTM	90.80
False	False	Tru	False	LSTM	94.68
True	False	False	True	LR	94.51
True	True	False	False	LSTM	92.12
False	True	True	False	LSTM	94.68
False	True	False	True	LR	94.51
True	False	True	False	LSTM	96.17
True	False	False	True	LR	95.50
True	True	True	False	LSTM	96.47
True	True	False	True	LR	95.72

**Table 18:** The mean cross-validation scores for DenseNet-201, Swin, HOG, and LSTM combination without CLAHE.

Deep Features	Fold	Accuracy (%)	Specificity (%)	Precision (%)	Recall (%)	F1-Score (%)	AUC (%)
Swin + DenseNet-201 + HOG + LSTM	1	96.94	91.20	97.35	91.20	93.55	99.14
	2	96.83	92.83	94.18	92.83	93.45	99.20
	3	97.73	95.04	96.64	95.04	95.78	99.33
	4	97.51	93.72	94.98	93.72	94.33	99.54
	5	96.94	91.86	96.95	91.86	94.18	99.04
	Average	97.19	92.93	96.02	92.93	94.26	99.25

**Table 19:** The pairwise approximate *p*-value matrix.

Backbone	Comparison	LSTM Mean (%)	Other Model Mean (%)	*t*-Statistic	*p*-Value	Significant?
Swin + DenseNet-201 + HOG	LSTM vs. LR	96.47	96.10	8.33	0.0012	Yes
	LSTM vs. SVM	96.47	94.43	24.68	0.0001	Yes
Swin + DenseNet-121 + HOG	LSTM vs. LR	96.20	95.33	21.45	0.0001	Yes
	LSTM vs. SVM	96.20	94.48	19.77	0.0001	Yes
Swin + MobileNet + HOG	LSTM vs. LR	96.45	95.90	16.84	0.0002	Yes
	LSTM vs. SVM	96.45	94.21	23.56	0.0001	Yes
Swin + VGG-16 + HOG	LSTM vs. LR	95.50	94.53	18.17	0.0001	Yes
	LSTM vs. SVM	95.50	92.67	28.32	0.0001	Yes

**Table 20:** The CI values for the performance metrics of the LSTM model.

Metric	Mean (%)	Std (%)	SE (%)	Margin of Error (%)	95% CI Lower (%)	95% CI Upper (%)
Accuracy	96.47	0.53	0.24	0.66	95.81	97.13
Specificity	91.76	1.46	0.65	1.81	89.95	93.57
Precision	94.92	1.19	0.53	1.48	93.44	96.40
Recall	91.76	1.46	0.65	1.81	89.95	93.57
F1-Score	93.15	0.80	0.36	0.99	92.16	94.14
ROC AUC	99.38	0.25	0.11	0.31	99.07	99.70

**Table 21:** The mean cross-validation scores for the four ML models with DenseNet-201, Swin, and HOG combinations.

Deep Features	ML	Accuracy (%)	Specificity (%)	Precision (%)	Recall (%)	F1-Score (%)	AUC (%)
Swin + DenseNet-201 + HOG	LSTM	87.16	85.51	86.72	85.51	85.78	98.61
	RF	81.03	78.97	80.47	78.97	79.37	95.86
	DT	62.64	61.98	61.98	61.98	61.82	78.69
	LR	86.38	85.73	85.39	85.73	85.46	98.39

**Table 22:** The classifier computational costs for different combinations of DL, Swin, and HOG.

Deep Features	Classifier	Accuracy	Total Training Time in sec	Total Inference Time in sec	Mean Inference Time per Sample in sec
Swin + DenseNet-201 + HOG	LSTM	97.15	23.50283131	0.115113512	2.61 × 10^−5^
	LR	96.69	71.82096811	0.030262081	6.85 × 10^−6^
	RF	90.06	87.64187354	0.371933951	8.42 × 10^−5^
	DT	81.49	166.3531168	0.009264886	2.10 × 10^−6^
	SVM	95.63	290.8598167	18.42127706	4.17 × 10^−3^
Swin + DenseNet-121 + HOG	LSTM	97.30	23.33571441	0.122186632	2.77 × 10^−5^
	LR	96.76	42.58101694	0.022082976	5.00 × 10^−6^
	RF	90.35	62.10678473	0.38177048	8.65 × 10^−5^
	DT	81.74	103.8163351	0.007941222	1.80 × 10^−6^
	SVM	95.36	179.6389052	11.06923762	2.51 × 10^−3^
Swin + MobileNet + HOG	LSTM	97.35	23.58124301	0.119072952	2.70 × 10^−5^
	LR	96.83	45.96610232	0.031669162	7.17 × 10^−6^
	RF	90.06	59.25471043	0.380624645	8.62 × 10^−5^
	DT	96.13	212.2095737	13.26019561	3.00 × 10^−3^
	SVM	82.99	118.5743992	0.008701353	1.97 × 10^−6^
Swin + VGG-16 + HOG	LSTM	96.58	26.54096419	0.128272788	2.91 × 10^−5^
	LR	96.47	126.2391764	0.072033748	1.63 × 10^−5^
	RF	88.79	61.24936068	0.421210498	9.54 × 10^−5^
	DT	81.72	147.2032004	0.015383337	3.48 × 10^−6^
	SVM	95.27	645.3330476	46.06173035	1.04 × 10^−2^

**Table 23:** The feature extraction cost breakdown for DenseNet-201, Swin, and HOG.

Feature	Time
Swin time in sec	108.16
Densenet-201 time in sec	139.40
HOG time in sec	4.11
HOG Reduction time in sec	0.67
Fusion time in sec	0.01
Total_feature_extraction_time_sec	4667.35

**Table 24:** The performance comparison with the state-of-the-art techniques.

Reference	Methodology	Performance	Datasets
Y.M. et al. [2]	Swin transformer, MobileNet-V2, and a bagging ensemble	Accuracy at 95.6%	X-ray imaging
Yüksel et al. [5]	DL framework	Precision at 89.4%	X-ray imaging
You et al. [6]	CNN	MioU of 0.736 ± 0.174	X-ray imaging
Baydar et al. [14]	U-Net architecture	Precision at 94.91%	X-ray imaging
Lee et al. [15]	CNN	AUC of 0.917 (95% CI 0.860–0.975)	X-ray imaging
Deng et al. [16]	CNN	Accuracy at 93.04%.	X-ray imaging
Abdalla-Aslan et al. [17]	A Cubic Support Vector Machine algorithm paired with Error-Correcting Output Codes	Accuracy at 93.6%	X-ray imaging
Ghaznavi Bidgoli et al. [18]	AlexNet and VGG-16.	Precision at 92%	X-ray imaging
Jaiswal et al. [19]	ResNet50-V2, ResNet101-V2, MobileNet-V3Large, MobileNet-V3Small	Accuracy at 93.2%	X-ray imaging
Krois, J. et al. [20]	CNN	SD of accuracy at 0.81	X-ray imaging
The proposed hybrid framework	Swin, DenseNet-201, and HOG	Accuracy at 96.47%	X-ray imaging

## Data Availability

We utilized the DRAD dataset. It can be accessed on Kaggle via this link: https://www.kaggle.com/datasets/imtkaggleteam/dental-radiography (accessed on 30 November 2025).

## References

[1] Arikan A., Özkan G., Pirinççi S., Abacıgil F., Sönmez I., Okyay P. (2019). Hekim Adaylarinin Ağiz-Diş Sağliği Alişkanliklari Ve Bilgi Düzeylerinin Değerlendirilmesi. Atatürk Üniversitesi Diş Hekim. Fakültesi Derg..

[2] Alsakar Y.M., Elazab N., Nader N., Mohamed W., Ezzat M., Elmogy M. (2024). Multi-Label Dental Disorder Diagnosis Based on MobileNetV2 and Swin Transformer Using Bagging Ensemble Classifier. Sci. Rep..

[3] Aldanma Ö., Atardağ H.B., Özdemir E.Y., Özyurt F. (2024). AI-Driven Dental Radiography Analysis: Enhancing Diagnosis and Education Through YOLOv8 and Eigen-CAM. TS.

[4] Mallya S., Lam E. (2019). White and Pharoah’s Oral Radiology: Principles and Interpretation.

[5] Yüksel A.E., Gültekin S., Simsar E., Özdemir Ş.D., Gündoğar M., Tokgöz S.B., Hamamcı İ.E. (2021). Dental Enumeration and Multiple Treatment Detection on Panoramic X-Rays Using Deep Learning. Sci. Rep..

[6] You W., Hao A., Li S., Wang Y., Xia B. (2020). Deep Learning-Based Dental Plaque Detection on Primary Teeth: A Comparison with Clinical Assessments. BMC Oral Health.

[7] Wang C.-W., Huang C.-T., Lee J.-H., Li C.-H., Chang S.-W., Siao M.-J., Lai T.-M., Ibragimov B., Vrtovec T., Ronneberger O. (2016). A Benchmark for Comparison of Dental Radiography Analysis Algorithms. Med. Image Anal..

[8] Mashayekhi M., Majd S.A., Amiramjadi A., Mashayekhi B. (2023). Radious: Unveiling the Enigma of Dental Radiology with BEIT Adaptor and Mask2Former in Semantic Segmentation. arXiv.

[9] Jader G., Fontineli J., Ruiz M., Abdalla K., Pithon M., Oliveira L. (2018). Deep Instance Segmentation of Teeth in Panoramic X-Ray Images. Proceedings of the 2018 31st SIBGRAPI Conference on Graphics, Patterns and Images (SIBGRAPI), Parana, Brazil, 29 October–1 November 2018.

[10] Kahurke S. (2023). Artificial Intelligence Algorithms and Techniques for Dentistry. Proceedings of the 2023 1st International Conference on Cognitive Computing and Engineering Education (ICCCEE), Pune, India, 27–29 April 2023.

[11] Sur J., Bose S., Khan F., Dewangan D., Sawriya E., Roul A. (2020). Knowledge, Attitudes, and Perceptions Regarding the Future of Artificial Intelligence in Oral Radiology in India: A Survey. Imaging Sci. Dent..

[12] Litjens G., Kooi T., Bejnordi B.E., Setio A.A.A., Ciompi F., Ghafoorian M., Van Der Laak J.A.W.M., Van Ginneken B., Sánchez C.I. (2017). A Survey on Deep Learning in Medical Image Analysis. Med. Image Anal..

[13] Dental Radiography Analysis and Diagnosis Dataset. https://www.Kaggle.Com/Datasets/Imtkaggleteam/Dental-Radiography.

[14] Baydar O., Różyło-Kalinowska I., Futyma-Gąbka K., Sağlam H. (2023). The U-Net Approaches to Evaluation of Dental Bite-Wing Radiographs: An Artificial Intelligence Study. Diagnostics.

[15] Lee J.-H., Kim D.-H., Jeong S.-N., Choi S.-H. (2018). Detection and Diagnosis of Dental Caries Using a Deep Learning-Based Convolutional Neural Network Algorithm. J. Dent..

[16] Deng L.Y., Ho S.S., Lim X.Y. (2020). Diseases Classification Utilizing Tooth X-Ray Images Based on Convolutional Neural Network. Proceedings of the 2020 International Symposium on Computer, Consumer and Control (IS3C), Taichung City, Taiwan, 13–16 November 2020.

[17] Abdalla-Aslan R., Yeshua T., Kabla D., Leichter I., Nadler C. (2020). An Artificial Intelligence System Using Machine-Learning for Automatic Detection and Classification of Dental Restorations in Panoramic Radiography. Oral Surg. Oral Med. Oral Pathol. Oral Radiol..

[18] Ghaznavi Bidgoli S.A., Sharifi A., Manthouri M. (2021). Automatic Diagnosis of Dental Diseases Using Convolutional Neural Network and Panoramic Radiographic Images. Comput. Methods Biomech. Biomed. Eng. Imaging Vis..

[19] Jaiswal P., Katkar V., Bhirud S.G. (2022). Multi Oral Disease Classification from Panoramic Radiograph Using Transfer Learning and XGBoost. Int. J. Adv. Comput. Sci. Appl..

[20] Krois J., Ekert T., Meinhold L., Golla T., Kharbot B., Wittemeier A., Dörfer C., Schwendicke F. (2019). Deep Learning for the Radiographic Detection of Periodontal Bone Loss. Sci. Rep..

[21] Huang G., Liu Z., van der Maaten L., Weinberger K.Q. Densely Connected Convolutional Networks. Proceedings of the 2017 IEEE Conference on Computer Vision and Pattern Recognition (CVPR 2017).

[22] Simonyan K., Zisserman A. (2014). Very Deep Convolutional Networks for Large-Scale Image Recognition. arXiv.

[23] Howard A.G., Zhu M., Chen B., Kalenichenko D., Wang W., Weyand T., Andreetto M., Adam H. (2017). MobileNets: Efficient Convolutional Neural Networks for Mobile Vision Applications. arXiv.

[24] Liu Z., Lin Y., Cao Y., Hu H., Wei Y., Zhang Z., Lin S., Guo B. (2021). Swin Transformer: Hierarchical Vision Transformer Using Shifted Windows. Proceedings of the 2021 IEEE/CVF International Conference on Computer Vision (ICCV), Montreal, QC, Canada, 11–17 October 2021.

[25] Dosovitskiy A., Beyer L., Kolesnikov A., Weissenborn D., Zhai X., Unterthiner T., Dehghani M., Minderer M., Heigold G., Gelly S. (2020). An Image Is Worth 16×16 Words: Transformers for Image Recognition at Scale. arXiv.

[26] Dalal N., Triggs B. (2005). Histograms of Oriented Gradients for Human Detection. Proceedings of the 2005 IEEE Computer Society Conference on Computer Vision and Pattern Recognition (CVPR’05), San Diego, CA, USA, 20–25 June 2005.

[27] Hochreiter S., Schmidhuber J. (1997). Long Short-Term Memory. Neural Comput..

[28] Cortes C., Vapnik V. (1995). Support-Vector Networks. Mach. Learn..

[29] Breiman L. (2001). Random Forests. Mach. Learn..

[30] Mao R., Shi X., Shi Z. (2025). A Decision Tree Classification Algorithm Based on Two-Term RS-Entropy. Entropy.

[31] Olowe K.J., Edoh N.L., Zouo S.J.C. (2024). Jeremiah Olamijuwon Comprehensive Review of Logistic Regression Techniques in Predicting Health Outcomes and Trends. World J. Adv. Pharm. Life Sci..

[32] Liu H., Simonyan K., Yang Y. (2018). DARTS: Differentiable Architecture Search. arXiv.

[33] Dawson L.G., Moore D.S. (2006). Excel Manual for Moore and McCabe’s Introduction to the Practice of Statistics.

[34] Cabitza F., Campagner A., Soares F., García de Guadiana-Romualdo L., Challa F., Sulejmani A., Seghezzi M., Carobene A. (2021). The Importance of Being External. Methodological Insights for the External Validation of Machine Learning Models in Medicine. Comput. Methods Programs Biomed..

[35] Han S., Mao H., Dally W.J. (2016). Deep Compression: Compressing Deep Neural Networks with Pruning, Trained Quantization and Huffman Coding. arXiv.

